# Integrated assessment of irrigation canal lining and covering impacts on groundwater dynamics in the central Nile Delta, Egypt

**DOI:** 10.1038/s41598-026-62338-0

**Published:** 2026-07-22

**Authors:** Huda M. Abd-Elrazak, Tamer A. Gado, Asaad M. Armanuos

**Affiliations:** Department of Irrigation and Hydraulics Engineering, Faculty of Engineering, Tanta University, Tanta, Egypt

**Keywords:** Canal seepage, Groundwater recharge, Canal lining, Canal covering, MODFLOW, Nile Delta, Environmental sciences, Hydrology

## Abstract

The rehabilitation of irrigation canals may reduce conveyance losses but also diminish recharge to shallow aquifers. One significant source of irrigation canal water loss is seepage. Determining the possible advantages of employing seepage reduction technology and materials requires quantifying the seepage losses in irrigation canals and pinpointing the locations where the seepage loss is most noticeable. However, in numerous regions of the world, seepage from unlined irrigation canals is a major source of groundwater recharge for the shallow aquifers, especially in dry and semi-arid locations like Egypt. The main aim is to assess the effects of canal lining and covering on groundwater recharge and water budget; a secondary aim is to compare numerical seepage estimates with empirical and analytical formulas. In this study, groundwater modeling was implemented utilizing MODFLOW, empirical formulas, and analytical equations were applied. The simulated groundwater level has been compared with the observed groundwater level in observation wells for model calibration. The relationships between Egypt’s central Nile Delta groundwater aquifer system and the irrigation canals in the Gharbia Governorate, considering six operating scenarios: (i) three scenarios of concrete lining of irrigation canals, (ii) three scenarios of covering open canals, (iii) estimate amount of seepage losses from the canals by empirical formulas, analytical equations to verify it with Groundwater Modeling System (GMS) software. The model’s findings demonstrated that the water budget was entirely dependent on the seepage from return flows from irrigated areas, which is the main source of groundwater recharge and supplies roughly 58.4% of the total inflow to the Quaternary aquifer. The seepage from irrigated canals was only a small part of the overall water budget. The leaking from the canal provides around 19.96% of the groundwater replenishment. The percentage of seepage to the canals discharge in the base case (no lining or covering) equaled 2.66%. The concrete lining of irrigation canals (Gharbia and Zefta Directorates canals lining scenario) resulted in a decrease in the aquifer recharge (from 602081 to 23921 m^3^/day). The percentage of seepage to the canals discharge in this scenario reduced from 2.66% to 0.105 %. The water budget analyses associated with converting the open irrigation canals to covering (Gharbia and Zefta directorates canals covering scenario) showed that the aquifer system’s canal leakage accounted for 0.06 m^3^/day. The percentage of seepage to the canals discharge in this scenario reduced from 2.66% to 0.0 %. Also, the results showed that the empirical formula, Molesworth & Yennidunia (0.24 x 10^9^) m^3^/year along with GMS software (0.22 x 10^9^) m^3^/year, had good agreement with the results. The outcomes demonstrate that improvements in irrigation conveyance efficiency are accompanied by reductions in groundwater aquifer replenishment. Therefore, canal rehabilitation strategies should be evaluated not only in terms of surface-water conservation but also with respect to their impact on groundwater resources. The outcomes of this research could be helped the decision makers and the stakeholders for achievement of proper water resources management and sustainable development goals (Egypt view 2030) in the central Nile Delta, Ghariba Governorate, Egypt.

## Introduction

In numerous regions of the world, particularly in arid and semi-arid countries like Egypt, one important form of recharge in the shallow aquifers is the leakage from unlined canals. Consequently, determining groundwater-surface water interactions in watershed is fundamental for recognizing and knowing the hydrological cycle and using water resources sustainably^1^. In hydrological specialty, groundwater and surface water systems have long been seen as distinct entities^2^, considering their diverse physical, chemical, and biological attributes^3^. Depending on the catchment characteristics, streams can either lose water to aquifers or gain water from them^4^. Groundwater and surface water systems interchange flow in two primary directions, which are ascribed to upwelling of groundwater to the surface (effluent conditions) [gaining] and stream water loss to the subsurface (influent conditions) [losing]^5,6^. Along a river reach, these exchanges vary spatially, with portions of stream water either receiving or losing groundwater flow, or a combination of the two^7^. Numerous elements influence the exchanges, such as the topography of the streambed, the hydraulic conductivity of the streambed deposits, stream curvature and groundwater gradient^8,9^. Maurer^10^ found that leakage from the Allerman canal at Pine Nut Creek, Nevada, had a direct impact on the groundwater level in an underlying aquifer. This study was among the first to look at groundwater recharge through the system of the surface canals. The simulations show that the maximum quantity that may be reserved during a five-year period is 3500 acre-feet of recharge, which caused water levels to rise over 70 feet close to the basin and approach the ground surface. The impact of interactions between groundwater and surface water on hydrologic budgeting along the upper Rio Grande in New Mexico was detailed by Fernald and Guldan^11^^,^ where the water table was raised due to seepage within a month of the channel starting to flow. When Abdulrazzak and Morel‐Seytoux^12^ looked into the recharge of groundwater from ephemeral streams in arid regions, they found that the primary source of aquifer recharge was infiltration through ephemeral stream beds. In Chile’s lower valley of the Cachapoal River, Arumí et al.^13^ examined the influences of canal seepage and irrigation loss on the groundwater interactions. The study found that groundwater was recharged by 52% from canal seepage and 22% from crop irrigation loss. Also, the study suggested that the agricultural production and the hydrological system will be influenced by changing irrigation system and the channel lining. According to the results of three-dimensional (3D) regional model, it is shown that the return irrigation water and the surface water seepage are the major sources of groundwater recharge in the Nile Delta (ND) groundwater aquifer system^14^. According to Morad and Abdel Latif^15^^,^ about 95% of the groundwater recharge between Borg El Arab and El Hammam, Egypt, came via canal seepage, with the remaining 5% coming from percolation. Awad and El Fakharany^16^ employed models of MODFLOW and MODPATH to confirm that the water logging problem was caused by the seepage from canal of Ismailia and the irrigation network, adding excessive water of irrigation. In Shahrekord University’s lab, Iran’s earthen canals in Boldaji, Borujen City, Chaharmahal, and Bakhtiari Province were simulated by^17^. According to the findings, seepage from the canal raised groundwater levels by 3.5 to 11 cm. Deng and Bailey^18^ used the global sensitivity analysis (GSA) using a MODFLOW code to investigate and quantify the source of high groundwater levels in northern Colorado’s irrigated stream aquifer systems. The outcomes demonstrated that a sealant-coated canal lining reduced seepage from irrigation canal to groundwater system by more than 50% and might decline the groundwater level by 1.5 to 3 meters over a five-year period. Wan et al.^19^ discovered the hydraulic interaction between the Molin River and groundwater,the findings showed that the Molin River was replenished by groundwater in the low-level reaches and received groundwater in the upper reaches. Armanuos et al.^20^ utilized a three-dimensional MODFLOW model to evaluate how the Grand Ethiopian Renaissance Dam (GERD) and various groundwater pumping scenarios could affect the groundwater levels in the ND aquifer. After calibrating the model to represent canal recharge and aquifer properties, they tested reduced canal water levels, increased pumping, and a combination of both. The results showed that higher pumping rates cause greater groundwater levels to decline than reduced canal levels, mainly due to a protective clay layer that limits canal seepage. The combined scenario led to the largest drawdown, highlighting potential risks for future climate change impacts in the region. 

Ref^21^. investigated the major challenges facing irrigated agriculture in Egypt’s Western Desert reclamation projects. The study categorized several management strategies, such as improving irrigation efficiency, cultivating water-efficient crops, reusing treated wastewater, and strengthening groundwater monitoring systems. The outcomes suggested that the long-term success of these measures depends on the level of acceptance and implementation by farmers in the affected regions. El-Ghandour et al.^22^ addressed water scarcity and the growing pressures on the freshwater resources by improving the design of irrigation canals to minimize the water losses. A particle swarm optimization (PSO) approach has been applied to determine cost-effective canal cross-sections whereas accounting for construction expenses, canal lining costs, and water losses from seepage and evaporation. Application of the suggested model to El-Sheikh Gaber Canal in North Sinai confirmed substantial cost savings of 28–41%, highlighting the potential of PSO as an effective alternative to traditional canal design procedures^23^. evaluated the potential impacts of climate change on future irrigation water requirements for major crops in Upper Egypt under the RCP 4.5 and RCP 8.5 emission scenarios. Using projected temperature and rainfall data together with the CROPWAT model, the research estimated future evapotranspiration rates and irrigation demands for several strategic crops. The outcomes indicated a gradual increase in net irrigation water requirements throughout the century, with greater increases occurring under the high-emission scenario. Gabr et al.^24^ investigated the impact of climate change on irrigation water requirements and sprinkler irrigation system efficiency in two arid regions of Egypt’s Western Desert. Through integration between CROPWAT 8 and WaterGEMS models, future evapotranspiration rates, crop water demands, and irrigation network requirements under multiple climate scenarios up to 2100 have been assessed. The outcomes displayed that rising temperatures are projected to substantially increase evapotranspiration and irrigation water needs in both Siwa Oasis and West Elminya, leading to greater pressure on available groundwater resources.

Shalby et al.^25^ assessed the capacity of the Moghra aquifer to support expanding agricultural development in Egypt using a calibrated FEFLOW groundwater model that incorporated observed water levels and GRACE-derived storage data. They simulated eight long-term pumping scenarios from 1000 wells over 100 years, with extraction rates between 800 and 1500 m^3^/day per well. Predicted drawdown ranged from 59 to 112 m, representing roughly one-fifth to two-fifths of the aquifer thickness. Climate change effects, including sea level rise and higher crop water demand, were also assessed,increasing withdrawals to meet future irrigation needs intensified drawdown by about 7.5%. A sustainable strategy was proposed based on gradually increasing abstraction to irrigate about 85,700 acres while limiting groundwater decline to no more than one meter annually.

Selim et al.^26^ assessed water seepage from lined irrigation canals using the validated Slide2 model under varying canal shapes, liner materials, and thicknesses. Statistical and artificial intelligence approaches-nonlinear regression (NLR), multilayer perceptron (MLP-ANN), and radial basis function networks (RBF-ANN)—were developed using SPSS and MATLAB to predict seepage rates. Findings indicated that higher liner permeability greatly increases water losses, whereas thicker linings significantly reduce them, while canal side slopes have little influence. The ANN models outperformed nonlinear regression, demonstrating superior accuracy and reliability, making them effective and efficient tools for estimating seepage losses in lined trapezoidal canals. Eltarabily et al.^27^ applied the Slide2 model to analyze interactions between canal water and groundwater and to quantify seepage from both unlined and lined irrigation canals. After confirming the model’s reliability for an unlined canal case, they simulated multiple scenarios to examine how groundwater table position, berm width, and liner characteristics influence water losses. Results showed that when groundwater levels were higher than canal water levels, seepage decreased due to flow entering the canal, whereas lower groundwater levels increased losses as water moved into surrounding soil. Groundwater position had little impact on lined canals, while wider berms reduced seepage mainly in unlined cases. Canal lining proved highly effective, cutting losses by nearly 100% on average, and greater liner thickness further enhanced seepage reduction. Eltarabily et al.^28^ used MODFLOW to simulate the surface-groundwater interaction for the Ismailia canal in Egypt in order to evaluate the effect of the canal liner on variations of the groundwater levels. An estimated 3.5 million m3/day, or around 21.6% of the canal’s total flow, seeped into the aquifer from the unlined canal. Through temperature monitoring, Lee et al.^29^ demonstrated how groundwater and stream water interact in the Haean basin of Korea. In many places, heat trace has been shown to be an effective technique for figuring out how surface water and groundwater interact^30^. Zhu et al.^31^ used a variety of tracer techniques to evaluate the groundwater-surface water interaction in the Lake Baiyangdian watershed of the Northern China Plain. Finding recharge to groundwater system sources and the major variables influencing it was the first stage. The impact of groundwater pumping on the adjacent surface water was then examined. Over-extraction of groundwater declined the depth of lateral inflows along the recharge boundary, and the stream leakage contributed between 14% and 75% of the canal’s length, making it a substantial component of the computed water budget for the study region. 

Ref^32–34^. evaluated the impacts of agricultural expansion on groundwater sustainability in Egypt’s Minia Governorate, where groundwater serves as the main source of irrigation water. Utilizing a calibrated MODFLOW model, the study predicted the future groundwater level declines under both current pumping conditions and planned land reclamation activities, revealing greater drawdowns with agricultural expansion but remaining within acceptable limits. The investigation also displayed that declining groundwater levels will substantially increase the energy essential for water abstraction, leading to higher demands for solar-powered pumping systems over time^32–34^. introduced an integrated framework combining the MODFLOW groundwater model with machine learning techniques to determine sustainable groundwater extraction rates in the Oligocene and Eocene aquifers of Minia, Egypt. The method assessed current and future climate conditions under RCP 4.5 and RCP 8.5 scenarios, viewing that groundwater levels are projected to decline progressively by the end of the century^35,36^. studied how the distance from the Nile River effects the groundwater quality in two agricultural regions west of Minia that depend on the Eocene aquifer. The evaluation displayed that groundwater closer to the Nile generally holds better quality and is more suitable for agricultural use, while groundwater from areas farther away needs treatment before being utilized for drinking or other purposes^32–34^. investigated the use of machine learning approaches to rapidly estimation irrigation water quality in the Eocene aquifer of Minia, Egypt, using easily obtainable field measurements and site characteristics. Several models were tested to predict key irrigation water quality indices, with Gaussian Process Regression (GPR) delivering the most reliable outcomes. The results indicated that parameters such as electrical conductivity and distance from the Nile River can successfully predict groundwater suitability for irrigation without requiring extensive laboratory analyses^32–34^. proposed the Irrigation Groundwater Viability Index (IGVI), a new method for assessing the economic feasibility of groundwater use in agriculture by combining groundwater quality and pumping depth into a single assessment framework. Using groundwater samples collected from agricultural reclamation areas in Minia Governorate, Egypt, groundwater resources according to their suitability and extraction costs has been classified. The outcomes showed that most reclaimed lands have moderate groundwater viability, representing the usefulness of IGVI as a decision-support tool for sustainable groundwater management, cost-effective irrigation planning, and long-term food security.

One of the main reasons for water loss is seepage from unlined irrigation canals. Seepage losses from irrigation canal systems have been and still are a major problem for irrigation districts and worldwide water supplies. However, a number of factors that affect the amount of canal seepage, such as the permeability of the soil layers and the canal bed, the depth of water in the canal, the kind of lining, the shape of the canal section, and the groundwater depth, can cause the water seepage losses to vary greatly^37–39^. A significant portion of the water diverted for agricultural use can be attributed to seepage from unlined canals. Several sources have predicted a wide range of seepage percentages, some of which are local to a given area. According to^40^, 17 percent of the water transported for irrigation demand in the United States in 1985 was lost as a result of seepage into shallow groundwater aquifers or evaporation. More than 40% of the diverted water quantity can be lost due to seepage in earthen canals in the central Rio Grande Valley of New Mexico, according to research by^41^. According to^42^, the evaporation loss is obvious when the water in canal is exposed to the atmosphere at surface. However, the evaporation losses are typically not as significant as seepage losses. It could be between 0.25 and 1% of the overall canal discharge. According to studies by^43^, n semi-arid regions, seepage losses may make up 20–30% of the flow in unlined clay canals. According to^44^ analysis of water losses in canals, seepage loss in irrigation canals accounts for the majority of water conveyance losses (98.37%), whereas evaporation losses account for just 1.3% of the entire stream. The canal seepage rate was estimated by^45^ and ranged from 9.76 to 20.39 cm/day. Between the channel head works and the crop fields, 40 to 50 percent of water is wasted, according to the estimations^46^. Therefore, calculation of the seepage losses from canals is a significant portion for sustainable management of water resources and land. The seepage losses can be measured directly or indirectly (estimated) using the hydraulic parameters of the soil and the boundary conditions, like the depth to the groundwater level, the water depth of canal, and cross-section of canal. Common methods used for estimating canal seepage losses mostly containing field experiments, analytical equations, empirical formulas and numerical modelling.

Despite the various estimates, it is certain that seepage prevents a significant quantity of water diverted for irrigation from ever reaching agricultural areas. Seepage losses not only deplete freshwater resources but also lead to salinization, waterlogging and contamination of groundwater^39^. According to^47–49^, over the years, continuing seepage from channels also the percolation losses from irrigation fields have caused water table in some areas to increase gradually to quite high levels, resulting in soil degradation in many areas. In addition, seepage reduces irrigation efficiency, increases the operational costs, and causes water shortages downstream. In order to effectively handle the water resources in the Valley and the ND, the Ministry of Water Resources and Irrigation (MWRI) in Egypt launched a nationwide 2-year initiative for Irrigation Canals Rehabilitation (ICR) in April 2020, decrease the seepage losses from the irrigation canals system into groundwater and restore cross-sections of canals to their designed sections and to address Egypt’s annual water deficit^50^. The canals rehabilitation is done using a concrete lining. The aim of national project is to line 20000 km of the 55000 km of Nile Valley canals. After the project completion, it is predicted that reduction in seepage loss of about 5 billion m^3^. Consequently, The primary goals were to assess how the irrigation canals system and the central ND groundwater aquifer interacted under six different operating scenarios: (i) three scenarios in which irrigation canals are lined with concrete, and (ii) three situations in which the groundwater table is shown to have changed before and after the lining and covering of the open canals processes for various places at various times and estimate the amount of seepage losses from the canals by empirical formulas, analytical equations and verify it with GMS software.

## Materials and methods

### Site and location of case study

**Fig. 1:**
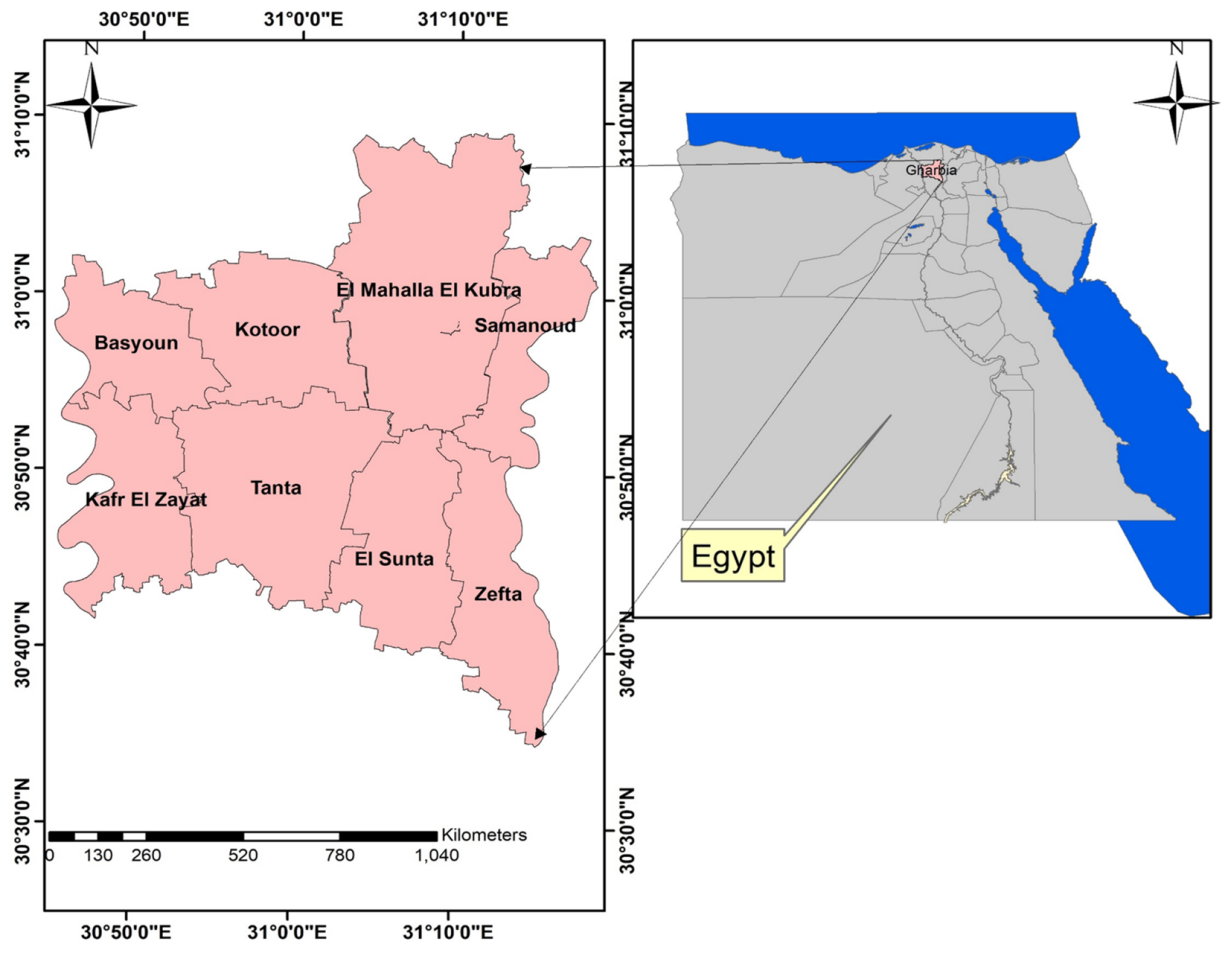
The Map of Gharbia governorate, the central of ND.

**Fig. 2:**
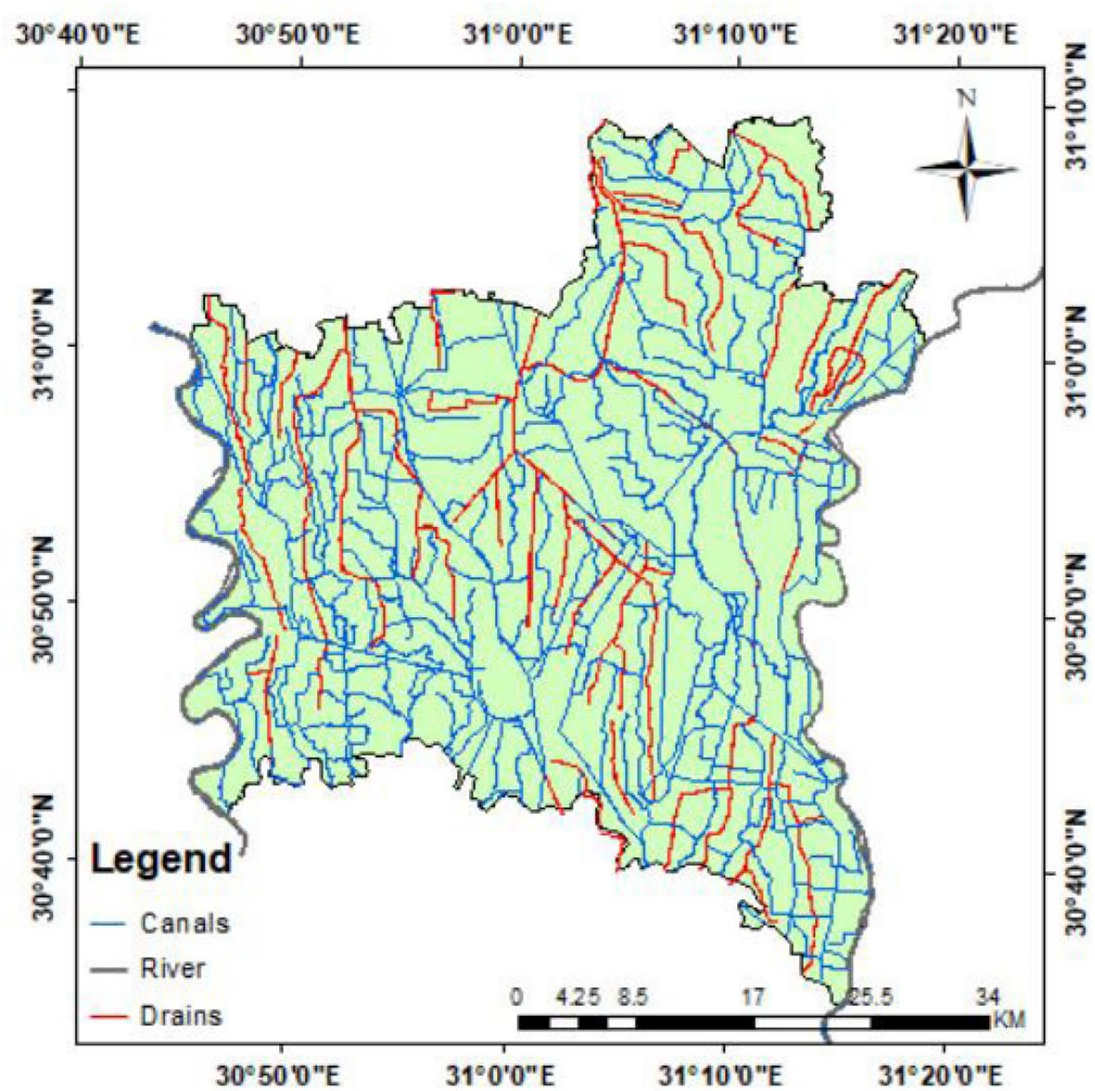
Surface water map in central part of ND region (Gharbia governate).

Gharbia governorate is situated in the middle region of ND, between latitude 30º 35ʹ, 31º 10ʹ N and longitude 30º 45ʹ, 31º 15ʹ E, as shown in Fig. 1. Its area is about 1942 km^2^ , where land uses vary between cultivated lands (1658 km^2^), residential lands (214 km^2^), and vacant lands (70 km^2^)^51^. It is bounded to the east by the Damietta branch and to the west by Rosetta branch. The governorate encompasses El-Menoufia in the south and Kafr-ELsheikh in the north. The total length of irrigation canals with a width of more than 25m is 212.8km, whereas irrigation canals with a width of 10 to 25 meters are 350.7 kilometers long overall. The surface water levels in these canals and drains generally increase in summer and decreases in winter, and the direction of water movement in the canals and drains is from south to north as shown in Fig. 2.

The topography generally slopes gradually northward from southward. The elevation of the surface is about 8.5m higher than the sea level in the south and gradually reduces to about 3.5m above sea level in the north. The slope coefficient in the province is about 10 cm/km^52^. In the middle region of the ND (Gharbia governorate), the quaternary sediments are composed of two hydrographic units: a layer of clay and silt from the semi-activated Holocene. Its thickness varies from 5 to over 20 meters. Sand and gravel with the clay lenses make up the lower Pleistocene unit. The Pliocene clay highlights the aquifer’s thickness, which ranges from 450 to 600 meters. The extent to which the surface water and the groundwater are hydraulically connected is significantly influenced by the lithological variations and thickness of the clay layer^53^. The Pleistocene aquifer’s groundwater levels vary throughout the Governorate, between 2 and 8 meters (above mean sea level) in the north direction and the south direction. It flows from the southeast to the northwest. In addition to seepage losses from the network of canals or directly from the Damietta branch, the aquifer system is constantly replenished by the infiltration of excess irrigation water. Rainfall could provide very little (negligible) recharge. Groundwater abstraction from current wells for irrigation and water supply, as well as natural outflow drains and the Rosetta branch, all contribute to aquifer discharge.

### Numerical simulation methodology

#### Groundwater flow equations

Darcy’s Law stated that " The flow of saturated water through a soil column is inversely correlated with column length and proportionate to head difference^54^".

Mathematically, it can be written as:

1 $$\mathrm{Q}=\mathrm{K}(\mathrm{A}\mathrm{d}\mathrm{h}/\mathrm{l} )$$

Where, Q is the discharge rate (m^3^/s), K is the aquifer hydraulic conductivity (m/s), A is the cross sectional flow area perpendicular to l (m^2^), and dh is the difference in head.

Groundwater from greater potential locations, such as recharge sites (greater height or increased hydraulic head or pressure), moves to places with lower pressure or lower elevation, according to the recognized hydraulic principle. This implies that the land surface’s topography should ideally determine the direction of groundwater flow. Rock materials are permeable due to fractures and linked pore spaces. Liquid can travel several meters per day for certain porous materials and several centimeters over a century for others. Groundwater flows in intricate three-dimensional patterns in the actual subsurface. One-dimensional and three-dimensional Darcy’s laws are comparable. The following partial differential equation describes the three-dimensional movement of groundwater across porous media when equation of Darcy’s flow the and the equation of continuity-which stands for the conservation of fluid mass-are combined:

2 $$\frac{\partial }{\partial \mathrm{x}}\left[{\mathrm{K}}_{\mathrm{x}\mathrm{x}}\frac{\partial \mathrm{h}}{\partial \mathrm{x}}\right]+\frac{\partial }{\partial \mathrm{y}}\left[{\mathrm{K}}_{\mathrm{y}\mathrm{y}}\frac{\partial \mathrm{h}}{\partial \mathrm{y}}\right]+\frac{\partial }{\partial \mathrm{z}}\left[{\mathrm{K}}_{\mathrm{z}\mathrm{z}}\frac{\partial \mathrm{h}}{\partial \mathrm{z}}\right]\pm \mathrm{W}={\mathrm{S}}_{\mathrm{s}}\frac{\partial \mathrm{h}}{\partial \mathrm{t}}$$

where, ${K}_{xx}$, ${K}_{yy}$, and ${K}_{zz}$ are the hydraulic conductivities for x, y, and z coordinates (L/T); h is potentiometric head (L); W is the volumetric flux per unit volume which expresses water sources and/or sinks. Positive sign refers to water entering the aquifer system from a source, on the other hand negative sign refers to water out from the aquifer system. It’s value less than zero when the flow out of the groundwater system, and it will be higher than zero when the flow is into the system (T^−1^); S_S_ is specific storage of porous media (L-1); and t is time (T).

#### Groundwater flow modelling

In this study, the numerical groundwater model GMS which is based totally on the MODFLOW model was applied. A conceptual modeling approach was used. The two main steps are the creation of the GMS model and the calibration using the measured groundwater levels. GMS was chosen for its adaptability and easy-to-use MODFLOW interface. MODFLOW version 2000 has been implemented through this study to investigate the impact of canals lining and covering on canals seepage and groundwater level decline. The model was run first for steady state for the year 2008, and for transient state from 2008 to the year 2020. The year 2020 is considered the base case, as the rehabilitation project started at the beginning of the year 2020. So, the six-management tested lining and covering scenarios started from the year 2020. The former involves the following steps:

- Import Arc GIS data and convert it to scatter data,
- Define boundaries,
- Create local sink / source package,
- Create stream package,
- Create recharge package,
- Define hydraulic conductivity,
- Create grid,
- Interpolate layer elevation,
- Convert conceptual model, and
- Simulation.

**Fig. 3:**
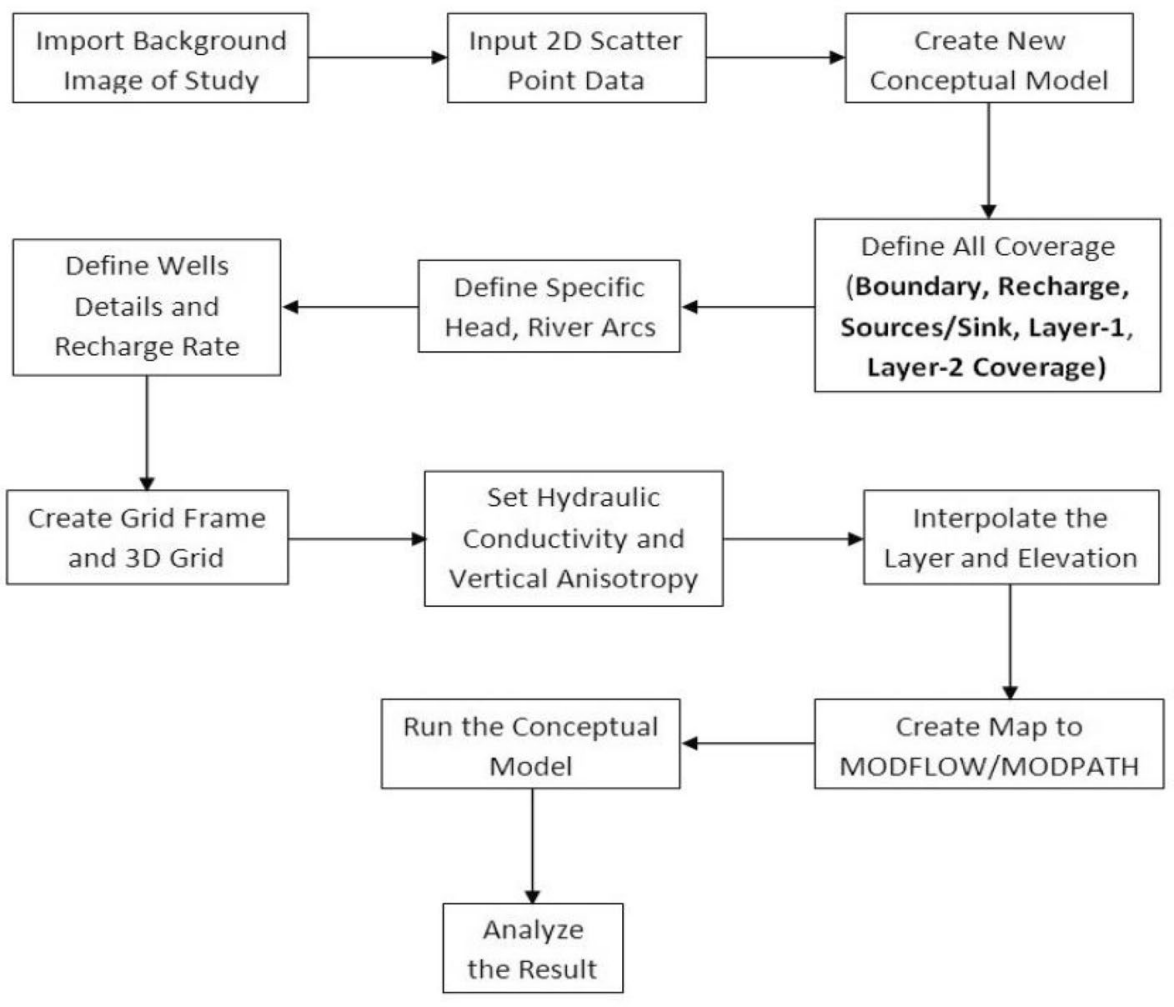
The Flow chart for development of GMS conceptual model.

Flow chart GMS conceptual model with the individual parameters collected and estimated from field data by the Ministry of Water Resources& Irrigation of Egypt is given in Fig. 3.

##### Boundary conditions

**Fig. 4:**
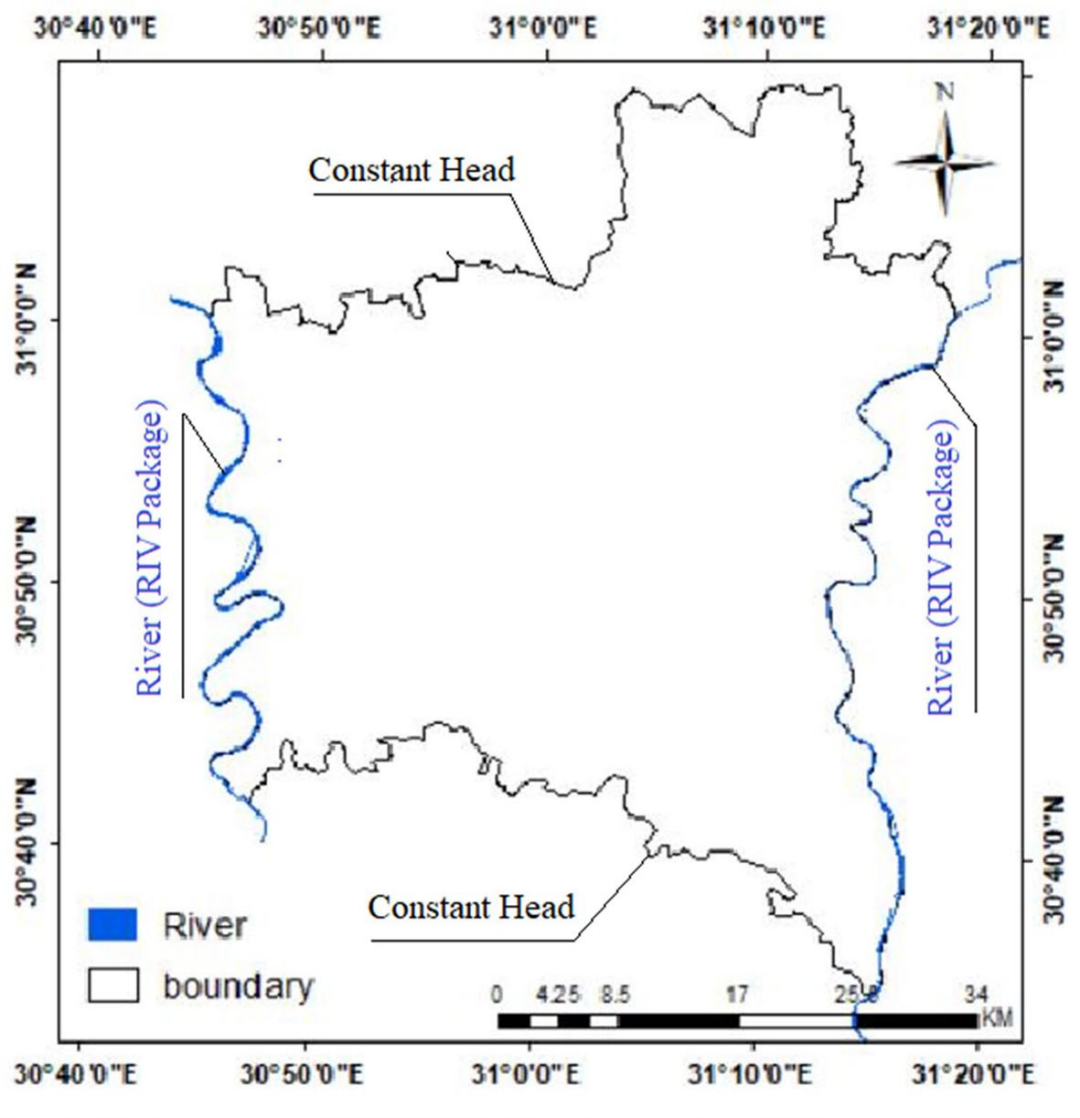
Conceptual model boundary conditions for the central Nile delta aquifer (Gharbia Governate).

Determining the model’s limits is the first stage in developing a conceptual model. Boundaries were developed from Arc GIS version using kml files created by Google Earth and imported into GMS. The middle region of the ND aquifer is represented by the research area. On the east and west sides of the model, distinct head borders are established. The eastern and western sides are both bounded by the Nile River. The model’s northern and southern regions were also identified as constant head boundaries, as shown in Fig. 4.

##### The Local source/sink package

Next, we defined the sources and the sinks package. Here, the research area’s boundaries and the wells’ locations were established.

##### (b-1) Creating the wells

**Fig. 5:**
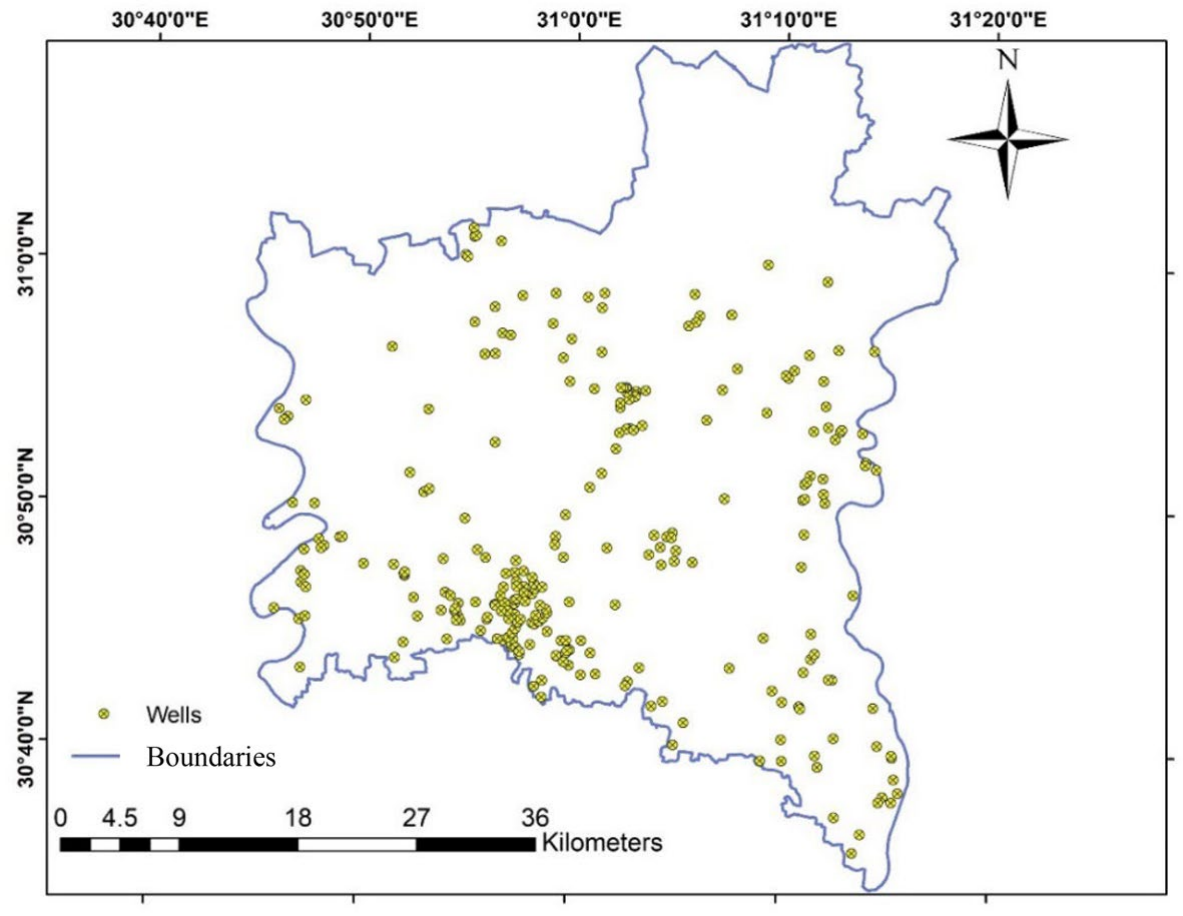
Distribution of groundwater wells in the central part of the ND, Ghariba Governorate and area boundaries.

In this study, wells that were not part of the study area were not included. A total of 265 abstraction wells were included in the simulation, as presented in Fig. 5. The data about the wells was loaded into GMS. from a text file. Every well has a refinement attributed to it. Due to the fact that wells represent points of convergence in groundwater flows, steep gradients occur near wells.

##### Recharge zone

**Fig. 6:**
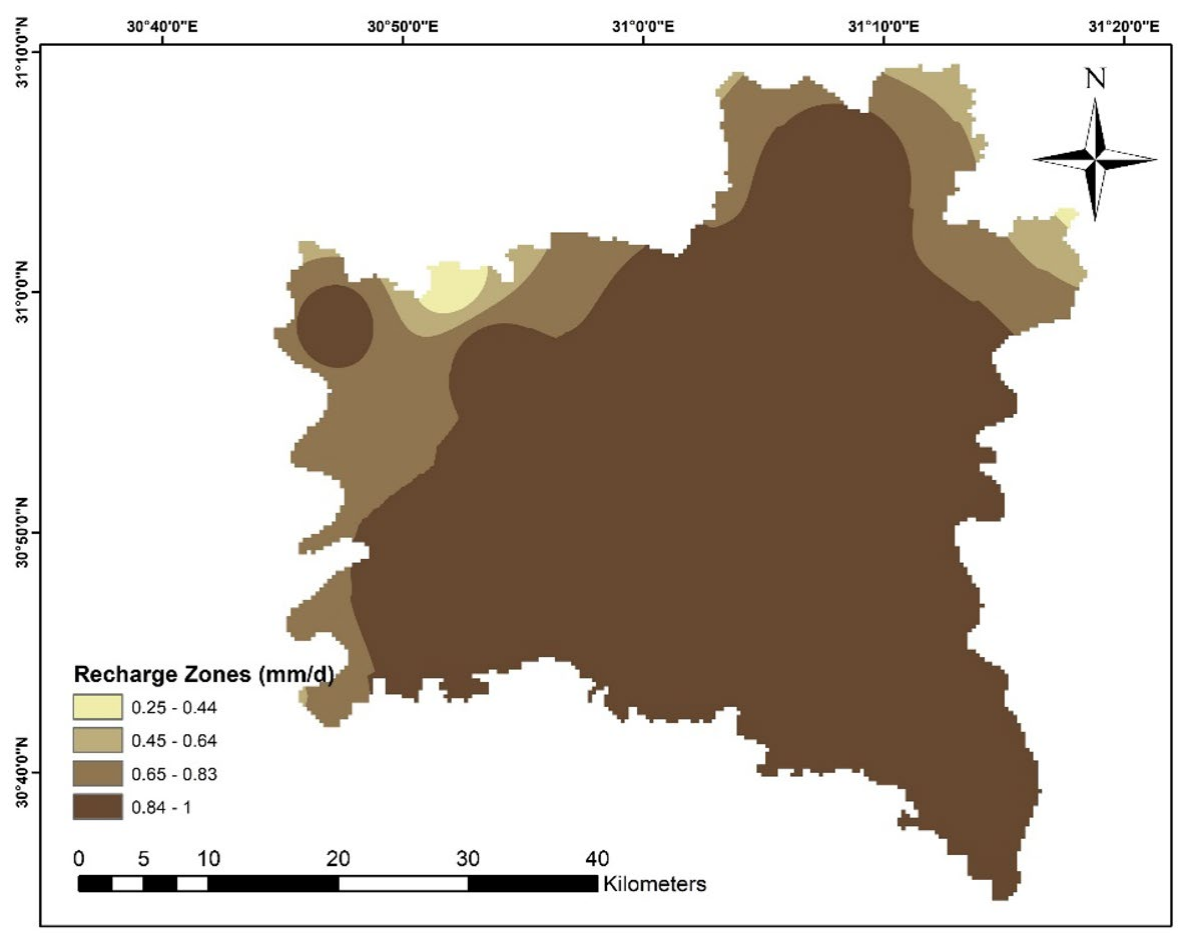
Distribution of groundwater recharge zones in the central ND, Egypt.

Determining the coverage of the recharging zones came next once the conceptual model was created. Recharge was thought to be consistent throughout the research region. It was believed that the irrigation water in GMS will supply the model with water replenishment. Fig. 6 shows recharge to the model and the initial value was set at 1 mm/day^55,56^. The steady state calibration used this value for the run of the steady state condition and the initial groundwater recharge rate.

##### Crop evapotranspiration

**Fig. 7:**
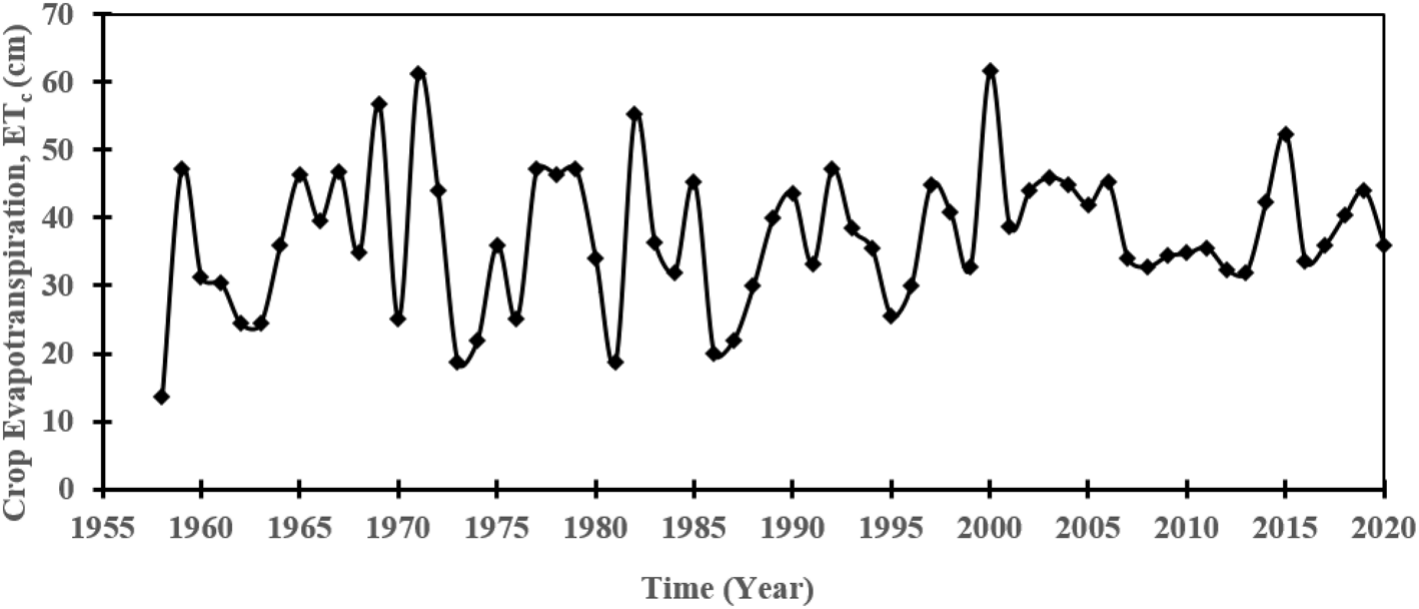
Historical data between 1958 and 2020 for the average ETc (cm) in the central of the Nile delta in the previous 62 years.

Agricultural water demand represents a major component of the hydrological system in the central Nile Delta and plays a crucial role in controlling groundwater recharge and irrigation requirements. Accordingly, accurate estimation of crop evapotranspiration (ET_c_) is essential for assessing water consumption patterns and evaluating the sustainability of water resources in the study area. In this research, ET_c_ was estimated using the Penman–Monteith method based on historical meteorological records and crop coefficients developed by the National Commission on Water Requirements. The analysis was implemented using wheat and maize, which are the predominant winter and summer field crops cultivated in the central Nile Delta. Wheat is counted Egypt’s primary strategic cereal crop, while maize is among the most extensively grown summer crops. Therefore, the combined water requirements of these crops provide a representative indicator of agricultural water use within the study area. Fig. 3 shows the long-term variation of average crop evapotranspiration in the central Nile Delta from the year 1958 to 2020. The results indicate that ET_c_ values fluctuated over the study period, displaying the impact of changing climatic conditions. Throughout the early years of the record, ET_c_ values generally varied between 20 and 35 cm, followed by several periods of increase and decline throughout the subsequent decades. Higher evapotranspiration values were observed during the late 1960s, early 1980s, and around the beginning of the twenty-first century, where ETc exceeded 50 cm in some years. After the year 2000, ETc exhibited moderate interannual variability, with values commonly varying between 35 and 50 cm, as shown in Fig. 7.

The observed fluctuations are primarily associated with temporal changes in meteorological variables such as air temperature, solar radiation, relative humidity, and wind speed. The overall pattern suggests a slight increase in crop water demand during recent decades, which may be attributed to climate variability and changing environmental conditions. These findings highlight the importance of improving irrigation efficiency and optimizing water allocation strategies to ensure sustainable agricultural production in the central Nile Delta.

##### Irrigation canals

**Fig. 8:**
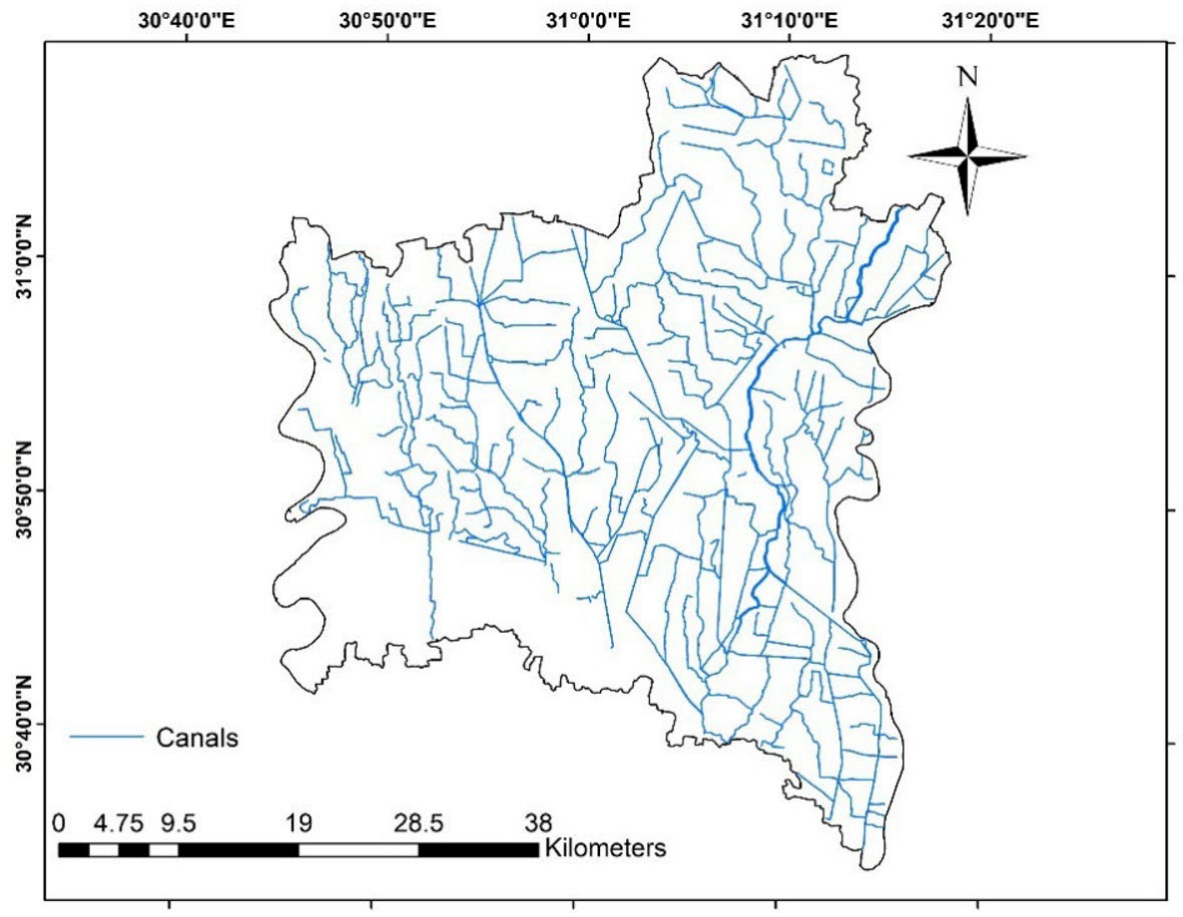
The Irrigation Canals in the central part of the ND, Ghariba governorate, Egypt.

Fig. 8 shows the irrigation canals in the area. These canals were modelled utilizing the STR (stream package) in the MODFLOW package. Canals in MODFLOW are identified as lines in the stream package. The STR package demands three inputs. First is the head stage in the canal (h). Secondly are top & bottom elevations. The third factor is the canal’s width, and the second is the conductance (C) values. The bed conductance is mostly determined by the canal infiltration resistance (the thickness of the bed x area/the value of the hydraulic conductivity), which is allocated branches that range from 50 to 100 m/day.

##### Hydraulic conductivity zone

**Fig. 9:**
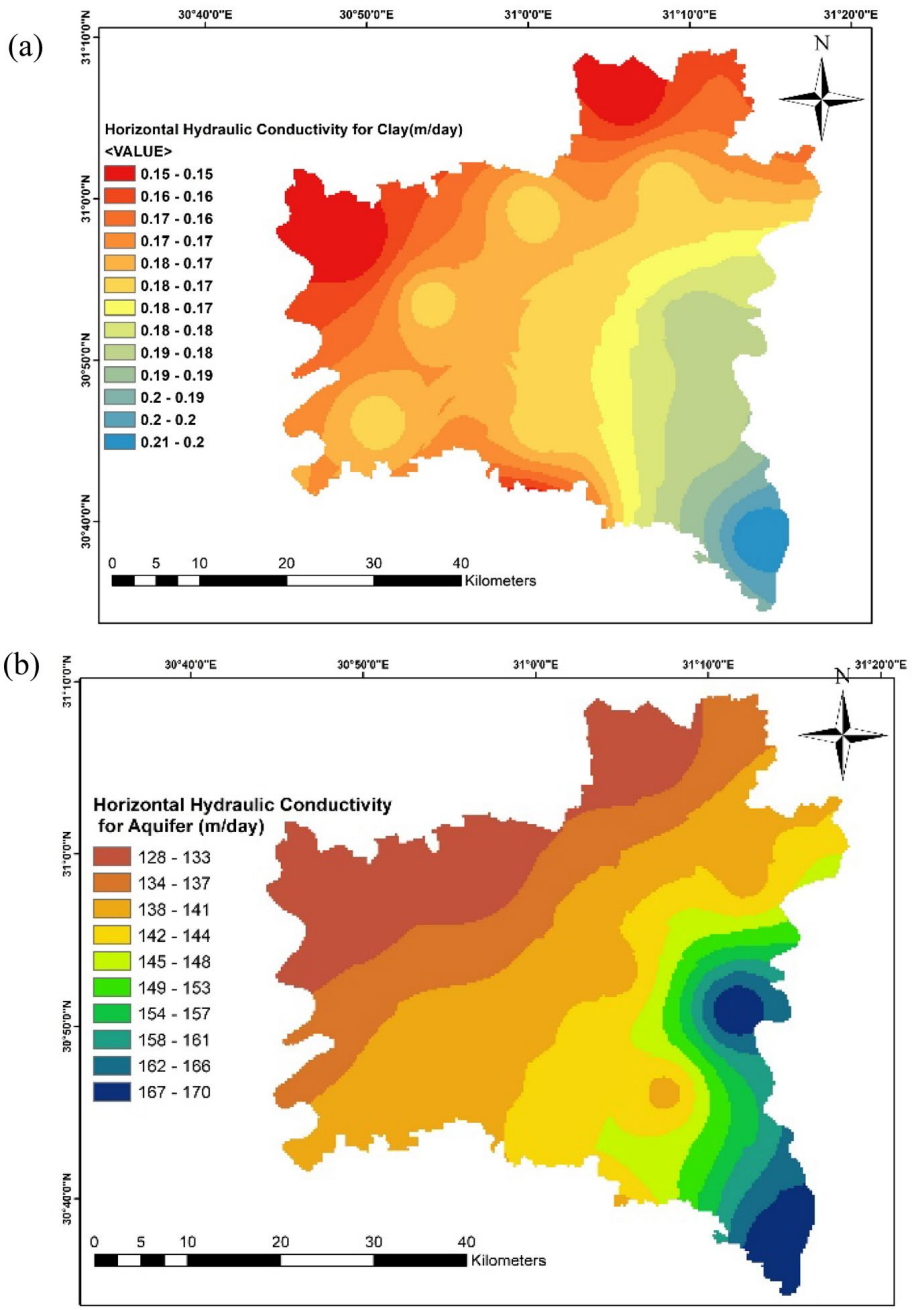
(**a**) calibrated vertical hydraulic conductivity of the first layer. (**b**) calibrated horizontal hydraulic conductivity of the second layer.

Setting up the hydraulic conductivity (K) coverage was the next step. For the steady state run, a number of polygons zones were defined for K. Fig. 9 (a) shows the calibration value of the horizontal hydraulic conductivity of the first layer in the central area of the ND region (Gharbia governate); it varies between 0.15–0.20 m/day for clay layer^56^. In the middle section of the ND (Gharbia governate), Fig. 9 (b) demonstrates the calibration value of the second layer’s hydraulic conductivity in the horizontal direction,it varies from 128-170 m/day^56^. Specific yields are defined inside this coverage. The value is set to 0.1^55^. The hydraulic conductivity distributions (Fig. 9) are based on the calibrated groundwater model of Armanuos^56^ and Armanuos et al.^20^. The maps were prepared by the authors using ArcGIS Desktop 10.8^57^ (Esri, Redlands, CA, USA) by clipping the Nile Delta model outputs to the Gharbia Governorate boundary with the Extract by Mask tool and applying graduated color symbology. Software: ArcGIS Desktop 10.8 Available at: https://desktop.arcgis.com/en/.

##### MODFLOW grid

**Fig. 10:**
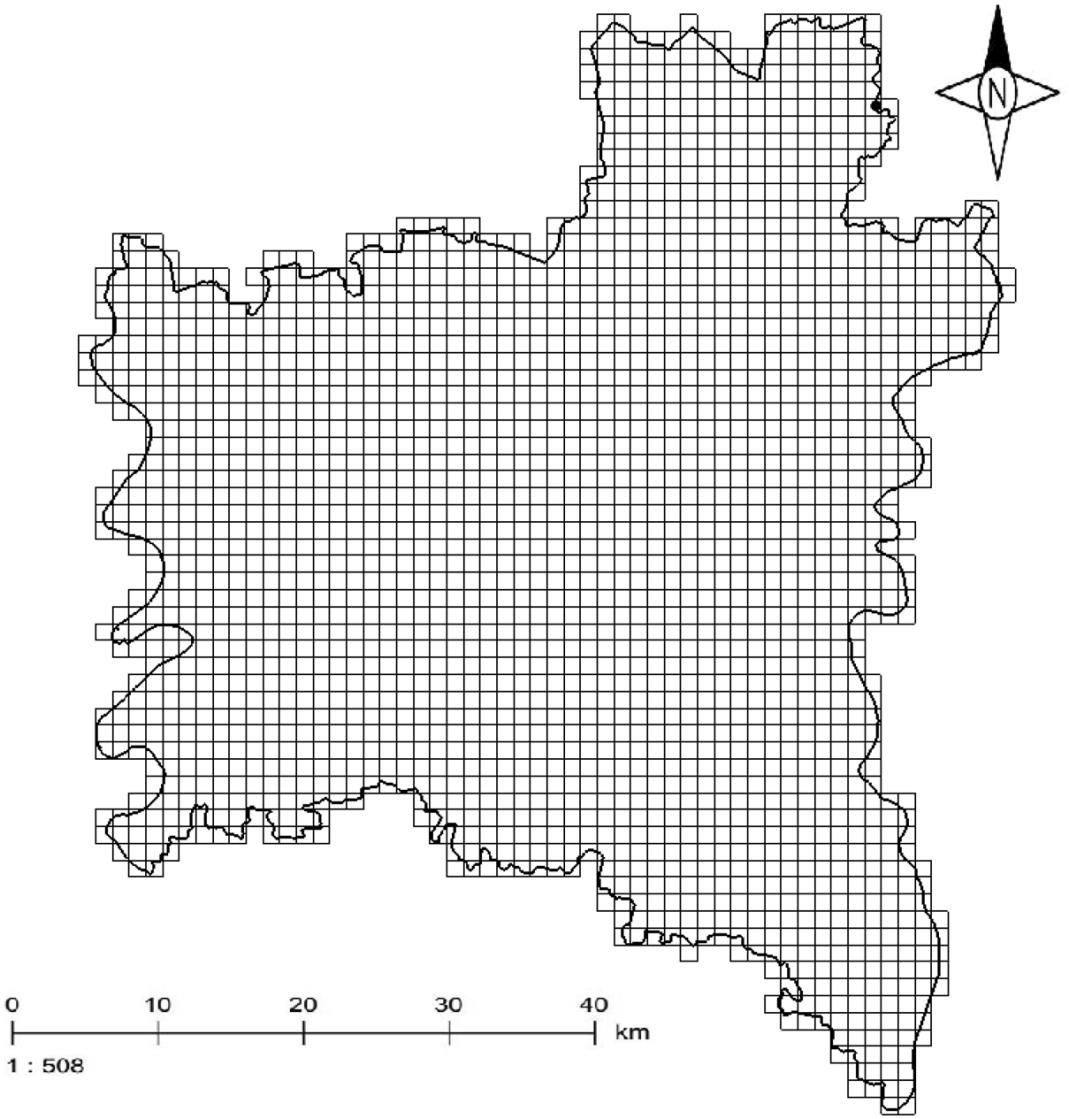
Model Grid of the Central part of the ND, Gharbia governorate, Egypt.

A groundwater flow model in the central areas of the ND region (Gharbia governate) was created using MODFLOW-2000 in GMS. Gharbia governate has an area of 1942 km^2^ and it is assumed to be rectangular in shape. Grid cells are designated as “active” within the model domain and “inactive” outside of it. The coverages are now set up and the MODFLOW grid has been produced. There are 56 discretized elements towards the x direction and 63 discretized elements towards the y direction within the model domain. Therefore, there were 2160 active cells in total 2160; each had a uniform cell size of 1000 m× 1000 m in the x and y directions respectively, as shown in Fig. 10.

##### Layer elevation

**Fig. 11:**
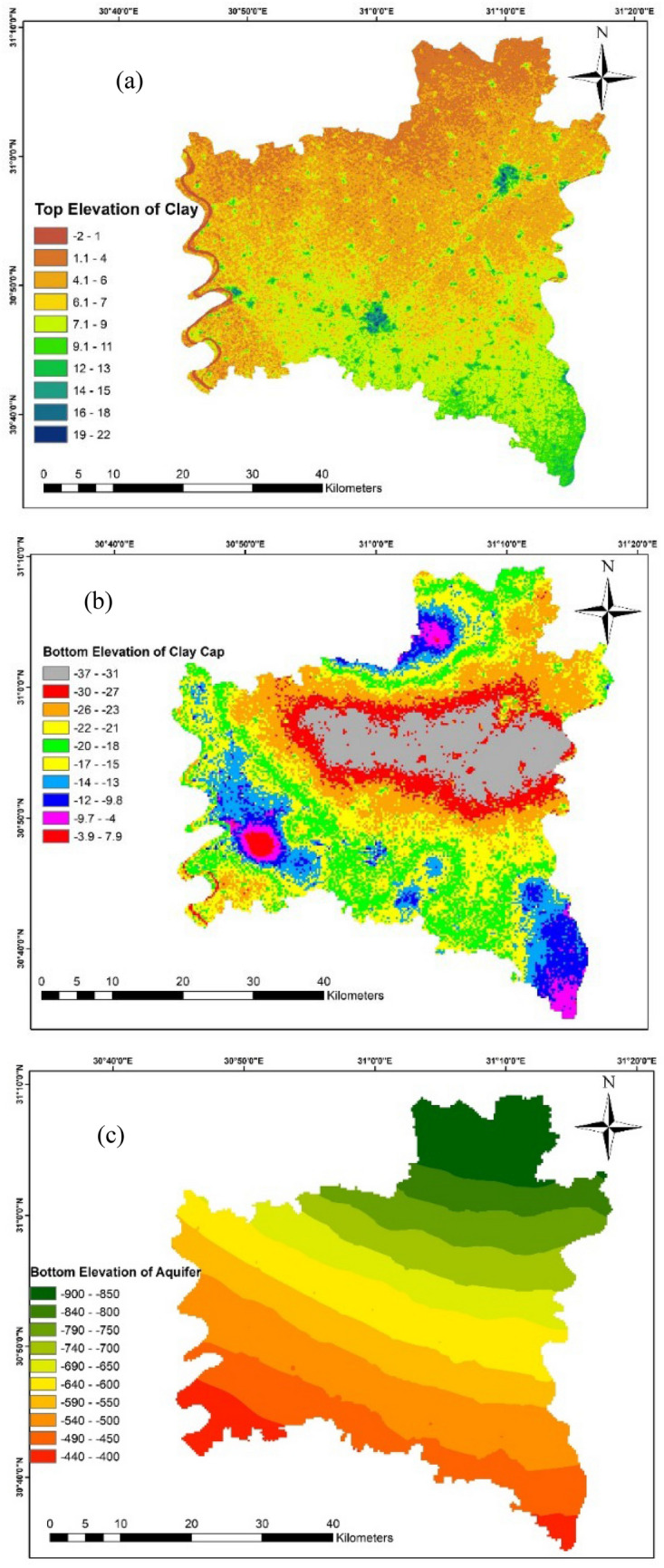
Arial Distribution of (**a**) Topography, (**b**) Base of clay cap and (**c**) Base of quaternary aquifer system.

Creating layer elevations is the final stage in finishing the conceptual model. A two-layer model was chosen for the study region to represent the hydrogeological conditions and the porous aquifer data at the study region. According to the DEM data obtained, the top elevation was computed. The MODFLOW layer of the built model interpolated this data. The clay cap’s highest elevation ranged from (−2) to (−22) m. The clay cap depth was utilized to interpolate the bottom elevation into the MODFLOW layer. The bottom elevation of clay cap varied from (8) to (−37) m, while the bottom elevation of aquifer varied from (-400) to (−900) m as shown in Fig. 11. Additionally, MODFLOW layers were interpolated from the initial head values. The model was run firstly for the year 2008 in the central area of the ND region (Gharbia governate to determine the hydraulic conductivities of sediments, conductance of the canals and infiltration rates from recharge. Groundwater levels which observed in the year 2008 were used as initial conditions of the model. As a next step, the built model was run under transient state condition between 2008 and 2020 in order to verify its accuracy, with a 1.5-meter head interval and using the water level data of the year 2020. The model’s goodness-of-fit was assessed with one set of measurements. A number of error-based measurements were made, including root mean squared error, R^2^, and mean absolute error based on comparison between the simulated groundwater level and the observed groundwater level on the year 2008 for steady-state condition, and the year 2020 for transient state condition. Moreover, correlation-based metrics were evaluated, such as the index of agreement, the efficiency coefficient, E, and the correlation coefficient, r, as shown in Fig. 11. Figure 11a presents the topography of the study area, Fig. 11b shows the base of the clay cap in Gharbia Governorate, and Fig. 11c displays the base of the Quaternary aquifer system. The maps were prepared by the authors using ArcGIS Desktop 10.8^57^ from spatial datasets obtained from the published Nile Delta groundwater model of Armanuos^56^ and Armanuos et al.^20^. Software: ArcGIS Desktop 10.8. Available at: https://desktop.arcgis.com/en/.

### Model calibration

The calibration of the built groundwater model was performed under steady state conditions and transient state conditions. Groundwater level data from 2008 was used for steady calibration, and observations from 2020 in the central ND region (Gharbia governate) were used for transient calibration. Using trial and error, the model was calibrated.

#### Methodology

Following model calibration, a water budget analysis was conducted to ascertain each parameter’s contribution to the model’s inputs and outputs. The water budget was computed by summing all inflows to and outflows from the groundwater system within the model domain. Inflows included irrigation return recharge, constant head boundaries (representing river leakage from the Damietta and Rosetta branches of the Nile River), and canal seepage. Outflows included discharge via constant head boundaries, pumping from production wells, and stream boundaries. The net flow between the aquifer and the irrigation distribution canals was calculated as the difference between total inflows and total outflows, representing the average daily seepage from canals to the aquifer system. The analysis was performed for both the steady-state calibration period (2008) and the transient base case (2020) to assess changes in the water budget over time.

#### Water budget – 2008 (steady state)

The water budget analysis for the year 2008 (Fig. 14a) showed that irrigation return recharge accounted for the largest proportion of total inflow, contributing approximately 69.8% of the total aquifer recharge. Constant head boundaries representing river leakage from both the Damietta and Rosetta branches of the Nile River contributed 14% of the total inflow. Canal seepage played a comparatively minor role, accounting for only 15.9% of the total aquifer recharge. Regarding outflows, approximately 98.95% of all outflows from the central portion of the Nile Delta aquifer occurred via constant head boundaries, while only 1.04% discharged through stream boundaries. The average net flow from distribution canals to the aquifer was calculated as 374,282 m^3^/day for the year 2008. Fig. 14a presents the spatial distribution of hydraulic heads for the year 2008 (steady state). The figure shows the head contours across the study area, with values ranging from approximately [2m] to [8m]. The head distribution reflects the natural groundwater flow direction from the Nile River branches toward the interior of the delta, with higher heads observed near the river boundaries and lower heads toward the center and northern parts of the domain. The steady-state heads served as initial conditions for the transient simulation and provided the baseline against which changes in head were later evaluated.

#### Water budget – 2020 (base case)

By the year 2020 (base case) (Fig. 14b), the water budget composition had shifted. Irrigation return recharge continued to dominate as the primary source of aquifer recharge, contributing approximately 58.4% of total inflow. Constant head boundaries representing river leakage from both Nile River branches accounted for 21.6% of total inflow, an increase compared to 2008. Canal seepage remained a minor component, providing only 19.96% of total aquifer recharge, though this represented a slight increase relative to 2008. Regarding outflows, pumping from the 265 production wells accounted for 29.5% of total outflows, while approximately 70.1% of outflows discharged through constant head boundaries and only 0.39% through stream boundaries. The average net flow from distribution canals to the aquifer increased to 590,230.81 m^3^/day for the year 2020 (base case), representing a significant increase compared to the 2008 value. Figure 15 shows the spatial distribution of hydraulic heads for the year 2020 (base case). The head contours illustrate the groundwater level distribution across the central part of the Nile Delta following the transient simulation from 2008 to 2020. Head values range from approximately [-4m] to [8m], with the highest heads concentrated near the river boundaries (Damietta and Rosetta branches) and the lowest heads observed in the interior and northern portions of the domain. The head distribution reflects the combined effects of irrigation return recharge, canal seepage, pumping, and boundary conditions.

#### Interpretation of dominant processes

The water budget analysis reveals several key insights regarding the groundwater system in the central Nile Delta region. First, irrigation return recharge is the dominant source of groundwater replenishment, consistently accounting for more than half of total inflows (69.8% in 2008 and 58.4% in 2020). This indicates that the aquifer system is heavily dependent on agricultural practices and irrigation water management for its sustainability. Second, canal seepage, despite being the focus of management interventions, represents only a minor component of the total water budget (15.9% in 2008 and 19.96% in 2020). This suggests that while canal seepage is not negligible, it is not the primary driver of groundwater recharge in the study area, and measures to reduce seepage may have a smaller impact on overall aquifer recharge than anticipated. Third, the increased contribution from constant head boundaries in 2020 (21.6%) compared to 2008 (14%) suggests enhanced hydraulic connection between the Nile River branches and the aquifer, possibly due to changes in water levels or aquifer conditions over time. Fourth, the net flow from canals to the aquifer increased substantially from 374,282 m^3^/day in 2008 to 590,231 m^3^/day in 2020, indicating an increasing contribution of canal seepage to groundwater recharge over the study period. Finally, the introduction of pumping from production wells as a significant outflow component (29.5%) in 2020 highlights the growing reliance on groundwater abstraction and its potential implications for long-term aquifer sustainability, emphasizing the need for careful groundwater resource management in the region.

### Water budget analysis

**Fig. 12:**
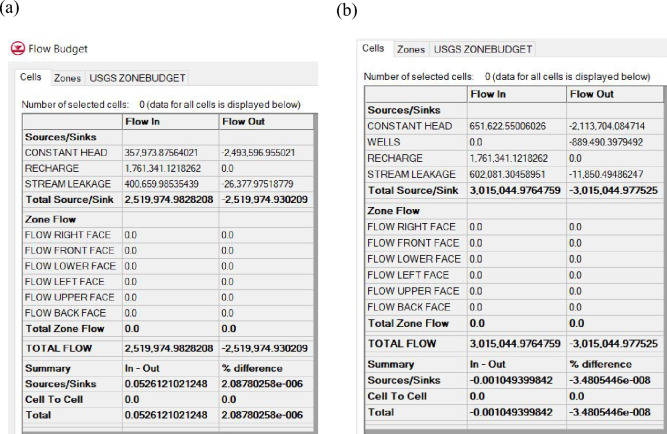
The values of water balance parameters after the model calibration of (**a**) the year 2008, (**b**) the year 2020.

A water budget analysis was conducted following model calibration to ascertain each parameter’s contribution to the model’s inputs and outputs. The biggest percentage of the entire inflow (69.8%) is accounted for by irrigation return recharge (Fig. 12a), whilst the constant head for river leakage on both Nile River beaches-the Damietta and Rosetta branches given in the built model simulation results-accounts for 14% of the total inflow. Just 15.9% of the total aquifer recharge comes from canal seepage. About 98.95% of all outflows came from the middle portion of the ND aquifer via constant head borders, with 1.04% coming via stream boundaries. Accordingly, in 2008, the average input-output flow from distribution canals to the aquifer was 374282 m^3^/day. The largest percentage (58.4%) of the entire inflow comes from irrigation return recharge, but the constant head for the river leakage on both beaches of the Nile River-the Damietta and Rosetta branches assigned in the model simulation-accounts for 21.6% of the overall inflow. Just 19.96% of the total groundwater aquifer recharge is due to canal seepage. About 70.1% of outflows went to the constant head borders, 0.39% went to stream boundaries, and 29.5% of all outflows came from the via pumping. Accordingly, in 2020 (base case), the average input-output flow from distribution canals to the aquifer was 590230.81 m^3^/day.

### Case scenarios

After that, four years of predictions were made from 2020 to 2024, with a different canal conductance assigned. After the proposed lining and covering canals were assigned, additional trials were tested through numerical simulations until the optimal discharge was obtained. Lastly, water budget calculations for the inflow and the outflow (recharge and discharge) characteristics were used to evaluate the effects of the canal lining and covering canals on the variations of the groundwater storage and sustainability. Table 1 presents the proposed scenarios for the canals. This study examined six potential outcomes that could impact the central portion of the ND aquifer. In accordance with the ongoing national canal restoration project in the ND region, the first three scenarios evaluate the effect of the canal lining on the groundwater aquifer system- irrigation canals interaction. In this rehabilitation, 15 cm concrete liners are placed over 30 cm dolomite stone layer. The studied canal lining scenarios are determined by reducing the canals’ hydraulic conductivity to 8.64X10^-5^ m/day. By enclosing the exposed irrigation canal segments in circular or rectangular conduits, the final three scenarios (hypothetical scenarios) examined the impact on the interaction. Covering the canals in the center of the Nile Delta resulted in reduction of the canals’ seepage losses and the canals evaporation losses from the open channels. Concrete closed conduits with a permeability of 8.64X10^-10^ m/day were suggested to cover the canals in these situations. The model’s assigned canal parameters, including the hydraulic conductivity, are shown in Table 2.

**Table 1:** The proposed scenarios for the canals.

No. of Scenarios	Canals lining scenarios			Covering canals scenarios		
	Scenario No 1	Scenario No 2	Scenario No 3	Scenario No 4	Scenario No 5	Scenario No 6
	Gharbia and Zefta directorates canals	Gharbia directorate canals only	Zefta directorate canals only	Gharbia and Zefta directorates canals	Gharbia directorate canals only	Zefta Directorate canals only
No. of canals	224	127	97	224	127	97
Length (km)	1182	707	475	1182	707	475

**Table 2:** Canals parameters for the proposed scenarios^58^.

Parameter	Canals lining scenarios	Canals covering scenarios
Hydraulic conductivity (m/day)	8.64 x10^−5^	8.64 x10^−10^

### Calculation of the canals’ seepage losses

In this study empirical formulas and analytical solutions were applied to calculate the seepage losses in different types of canals. Empirical formulas and analytical solutions were applied when direct canal measurement wasn’t possible. They’re based on relationships between the hydraulic conditions and the water losses. Several formulas were created to estimate for very localized, specific conditions, Conversely, some were made to forecast more general conditions (for example the lined canals or the unlined canals); others necessitate information on the canal’s discharge or the canal velocity or the soil’s saturated permeability^59,60^. According to Addams^61^ states that the following factors are known to have particular effects on leakage rate: permeability, porosity, texture, top width, duration of operation, age of the canal, canal wetted perimeter, dimensions/geometry, water depth in the canal, depth to ground water, soil and water temperature, salinity of the water and soil, sediment load in the canal, and others. The following Table 3 summarizes the most well-known formulas for calculating seepage losses and the parameters that affect seepage.

**Table 3:** Popular seepage equations.

NO.		Name of equations	Equations	Parameters	Affecting governing parametrs	Notes
1	Empirical formulas	Davis and Wilson	$S=0.45Cx\frac{PL}{4x{10}^{6}+3650{V}^{0.5}}x{h}^{0.5}$	(S) is seepage loss amount ( m^3^/sec), (P) is wetted perimeter ( m), (L) is canal section length ( m), (h) is water depth ( m), (V) is flow velocity in the canal (m/s)	Wetted Perimeter, Length, Water Depth, and Velocity	(C) is value of the numerical coefficient which depends on lining or soil type, c=1,4,5,12,25 and 70 for Concrete,Mass clay,Light asphalt ,clay soil,sandy loam,and coarse sand and gravel
2		The Egyptian formula	$S=CPL{R}^{0.5}$	(S) is seepage loss amount (m^3^/sec), (P) is canal wetted perimeter ( m), (L) is canal length (km), (R) is hydraulic radius (in m) = (A / P), and (A) is surface area that has been wet (m^2^)	Wetted Perimeter, Length, Hydraulic Radius,and Area	(C) is a numerical value that ranges from 0.0015 for clay soils to 0.0030 for sandy soils
3		Pakistanian formula	$S=\frac{5{PLQ}^{0.0652}}{{10}^{6}}$	(S) is seepage loss amount (in ft^3^/sec), (Q) is channel discharge (ft^3^/sec), (P) is perimeter that has been wet of channel (ft), and (L) is channel length (ft)	Wetted Perimeter, Length and Discharge	
4		Hungarian formula	$S=1700H{d}_{a}x\left(b+H{S}_{o}\right)$	(S) is seepage loss amount (in m^3^/day/per meter of length), (H) is canal water depth (m), (da) is the diameter of the soil grains’ effective size, (b) is canal bed width (m), and (So) is canal bottom slope	Soil Type, Length, Water Depth, Width,Slope, and Discharge	
5		Equation of Mowafy	$S=CAD$	(S) is seepage loss amount in the channel (cusec), (A) is channel area that has been wet (in ft^2^), (D) is channel water depth (in ft),	Water Depth,and Area	(C) is Constant value which Northern Indus plain’s C value ranged from 1.1 to 1.8,
6		Moritz equation	$q=0.0186C{\left(\frac{Q}{V}\right)}^{0.5}$	(q) is a value that represents the one-kilometer channel seepage rate (m^3^/s), (Q) is the quantity of water leakage from the canal (m^3^/s), and (V) is water velocity in channel (m/s).	Discharge, and Velocity	(C) is a constant coefficient ranging from 0.41 for clay loam and clay soils to 0.66 for sandy loam soils
7		Ingham’s equation	$q=0.55CPL{H}^{0.5}x{10}^{-6}$	(q) is seepage loss amount (m^3^/s), (P) is perimeter that has been wet of channel ( m), L is channel length (m), and (H) is depth of the water in the channel (m)	Wetted Perimeter, Length, and Water Depth	(C) is the coefficient, which varies according to soil type which ranges between 1.5 and 5.5,
8		Nazir Ahmed’s equation	$S=\frac{0.04{Q}^{0.68}}{56.81}$	(S) is seepage loss amount (m^3^/s/km), and (Q) is channel discharge (m^3^/s)	Discharge	
9	Analytical equations	Molesworth and Yennidunia equation	$S={a}_{s}Q{a}_{s}=\frac{0.375x{10}^{-4}}{{R}^{1.166}x{i}^{0.5}}$	(S) is seepage loss amount (m^3^/sec/km), (Q) is the canal discharge ( m^3^/sec), (R) is hydraulic radius (m ) = (A / P), (P) is perimeter that has been wet of canal ( m), (A) is canal area that has been wet ( m^2^ ), and (i) is bottom slope	Wetted Perimeter,Discharge,Slope, Length, Hydraulic Radius,and Area	

## Results and discussion

### Model simulation and calibration

**Fig. 13:**
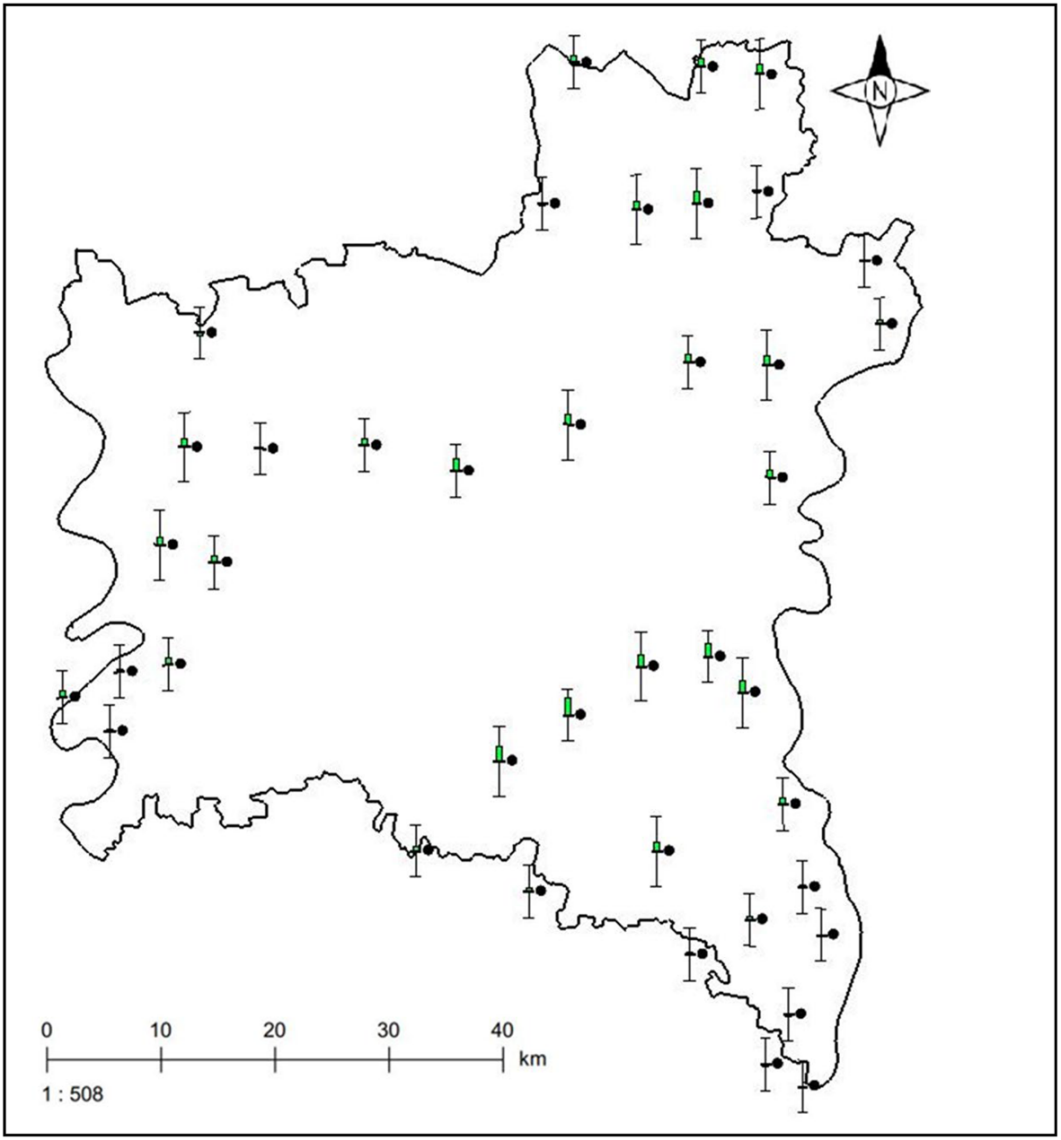
Distribution of observed groundwater wells in the central part of the ND, Ghariba Governorate and area boundaries.

**Fig. 14:**
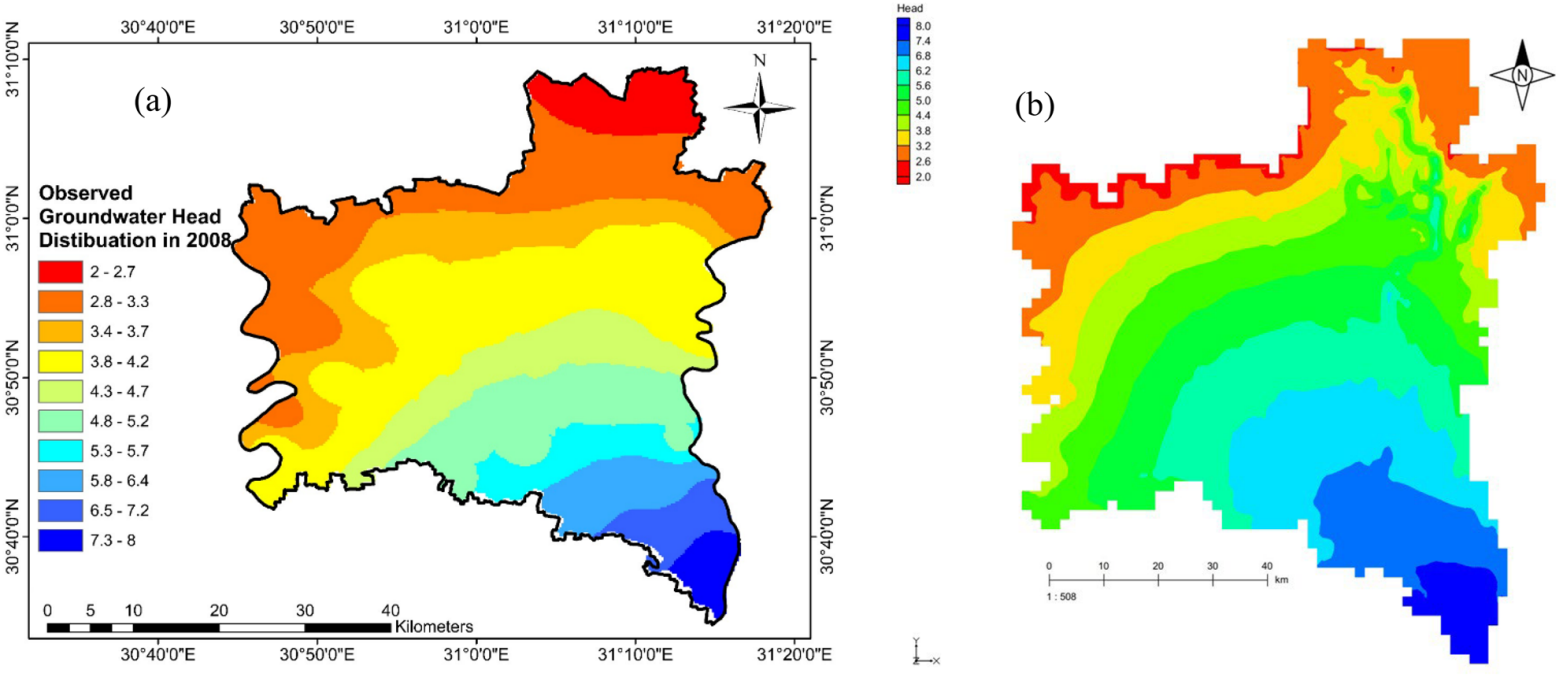
Comparison between the the observed groundwater head (**a**) for the year 2008 and the simulated groundwater head (MODFLOW) (**b**).

**Fig. 15:**
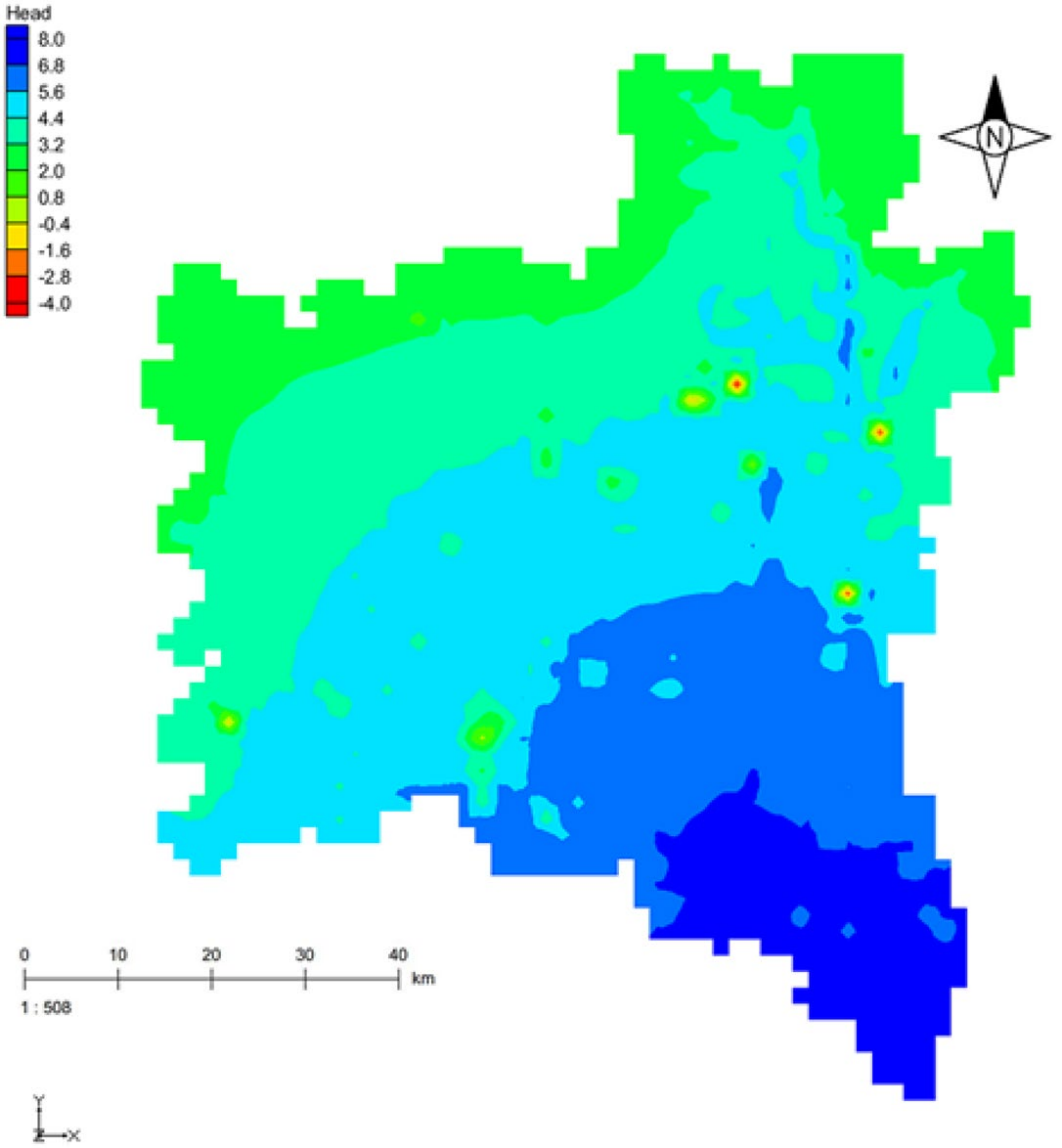
The groundwater heads distribution in the central part of the ND Region (Gharbia governate) at base case for the year 2020.

**Fig. 16:**
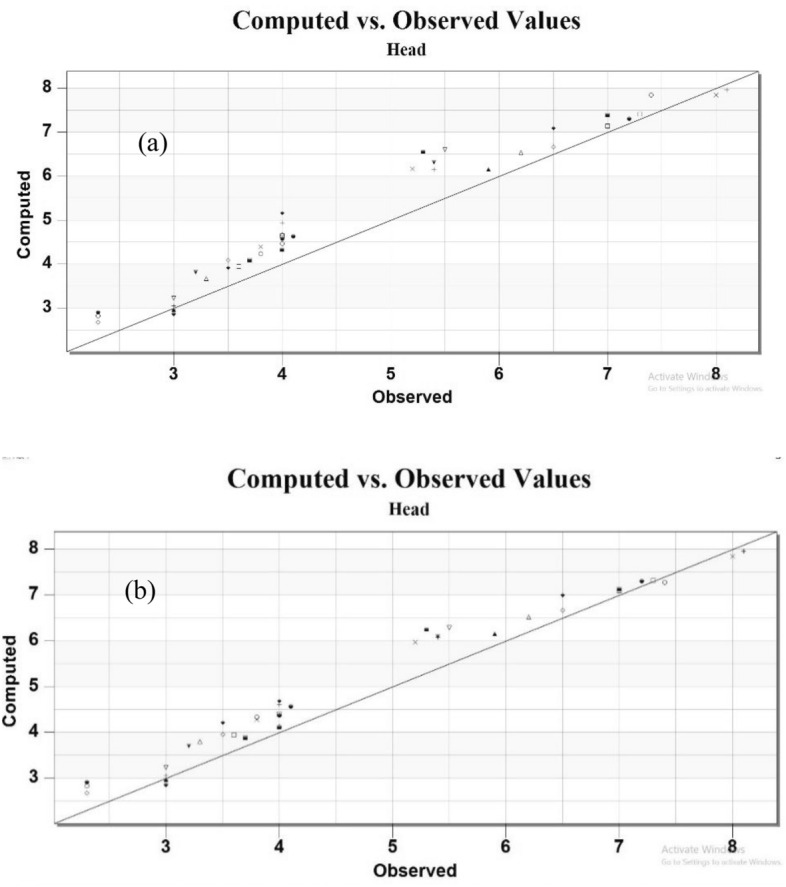
The observed vs. the calculated groundwater heads (m) (**a**) for steady state condition (2008), (**b**) for transient state condition (2020).

A conceptual built model of the central region of the ND aquifer was created, beginning and boundary conditions were assigned, and the built model was run for 12 years, from 2008 to 2020 (2008 for steady-state models and from 2008 to 2020 for transient models). Following a twelve-year calibration period between 2008 and 2020, the model was then run while taking the lining’s deployment into account in 2020. Despite the model’s one-year time step, the simulation compared groundwater heads twelve years later to groundwater measurements from 40 observation wells in the year 2008 and the year 2020, as shown in Fig. 13. Figure 14 presents a comparison between the simulated groundwater head from MODFLOW and the observed groundwater head for the year 2008. The presented groundwater head ranges from 2 to 8 m in the year 2008. Groundwater flows northward from the south, towards the Mediterranean Sea direction. A good correlation was found between the groundwater head observed and the simulated groundwater head distribution in the central area of the ND for the year 2008. Figure 15 shows the distribution of groundwater head in the central area of the ND, Ghariba Governorate, Egypt for the year 2020. From south to north, the groundwater head rose, and the flow of groundwater was directed northward toward the Mediterranean Sea. The groundwater head in the year 2020 ranges from 0.8 m and increased towards the south direction to reach 8 m, as shown in Fig. 15. Figure 16 shows the comparison between the simulated groundwater head and observed groundwater head for 40 different observation wells, for the years 2008 and 2020 for steady state condition and transient state calibration respectively. A good correlation between the observed groundwater head and the simulated groundwater head in the study area. Good agreement between field observations was demonstrated by the descriptive statistical analysis of the observed (measured) and computed (from the built model findings) potential heads. Model with RMSE=0.62 for the year 2008 and with RMSE= 0.44 for the year of 2020. The correlation and error-based parameters for this model calibration.

According to Fig. 15 at base case (current situation), The base case presents the current situation the central area of the ND aquifer, it is evident that the distribution canals’ net recharge reached 602081.3 m^3^/day. On the other hand, 11850 m^3^/day of groundwater flowed from the aquifer to the canals. The aquifer’s net outflow to the Rosetta and Damietta branches, which have constant head limits, was 2113704 m^3^/day. In 2020, the simulated groundwater level in the center portion of the ND (Gharbia governate) was 0.8 meters in the north and rose to 8 meters in the south. The groundwater level drops to 3.2 meters in the west, 4.4 meters in the southwest, and 2 meters in the northwest. Groundwater flows from the south direction towards the north direction.

### Water balance of the central part of ND aquifer

Table 4 summarizes the water budgets of the central ND aquifers. According to the base case scenario, which depicts the current state of the middle ND aquifer, the net groundwater recharge from the distribution canals has reached 602081.3 m^3^/day. On the other hand, 11850 m^3^/day of groundwater flowed from the aquifer to the canals. The aquifer’s net outflow to the Rosetta and Damietta branches, which have constant head limits, was 2113704 m^3^/day. In 2020, the simulated groundwater level in the central region of the ND (Gharbia governate) was 2 meters in the north and rose to 8 meters in the south. The groundwater level drops to 3.2 meters in the west, 4.4 meters in the southwest, and 2 meters in the northwest. As seen in Figure 15, the groundwater flows from the south direction towards the north direction.

**Table 4:** Zone budget analysis for the base case (year of 2020) and the six analysed scenarios in the central part of the ND region (Gharbia Governate) of canals lining and canals covering (values in m^3^/day).

Budget	Components	Scenarios number	Scenario No 1	Scenario No 2	Scenario No 3	Scenario No 4	Scenario No 5	Scenario No 6
	Boundary parameters	Base case	All canals lining	Gharbia canals lining	Zefta canals lining	All canals covering	Gharbia canals covering	Zefta canals covering
Inflow	Constant head	651623	736150	684804	700169	739820	684451	702134
	Wells	0	0	0	0	0	0	0
	Recharge	1761341	1761341	1761341	1761341	1761341	1761341	1761341
	Stream leakage	602081	23921	203881	431408	0	201801	423501
	Total inflow	3015045	2521444	2650050	2892935	2501188	2647617	2886993
Outflow	Constant head	2113704	1631586	1755926	1996828	1611698	1753672	1991069
	Wells	889490	889490	889490	889490	889490	889490	889490
	Recharge	0	0	0	0	0	0	0
	Stream leakage	11850	368	4633	6617	0	4455	6433
	Total out flow	3015045	2521444	2650050	2892935	2501188	2647617	2886993

#### Scenario 1: All canals lining (Gharbia and Zefta areas)

Under the all canals lining scenario in Gharbia Governorate, significant reductions in canal seepage were detected throughout the study area. Groundwater recharge from stream leakage declined dramatically from 602,081 m^3^/day in the base case to 23,921 m^3^/day, indicating a reduction of approximately 96%. Consequently, the total groundwater inflow declined to 2,521,444 m^3^/day, compared with 3,015,045 m^3^/day under current conditions. The reduced recharge resulted in a lower discharge through the constant head boundaries, which decreased from 2,113,704 m^3^/day to 1,631,586 m^3^/day. The groundwater contribution back to the canal network was also significantly reduced, reaching only 368 m^3^/day. These results of the current scenario show that comprehensive canal lining successfully minimizes seepage losses but substantially decreases aquifer recharge, thereby reducing groundwater storage and hydraulic gradients within the central Nile Delta aquifer system.

#### Scenario 2: Gharbia canals lining

The implementation of canal lining within the Gharbia area only produced moderate reductions in the rate of groundwater recharge. Canal seepage reduced to 203,881 m^3^/day, demonstrating a reduction of about 66% relative to the base case scenario. Total aquifer inflow declined to 2,650,050 m^3^/day, while discharge through the constant head boundaries decreased to 1,755,926 m^3^/day. Groundwater outflow from the aquifer system toward the canals was reduced to 4,633 m^3^/day, compared with 11,850 m^3^/day in the base case scenario. The results suggest that lining canals in Gharbia area only effectively reduces seepage losses while maintaining a larger proportion of groundwater recharge than the full-lining scenario. Consequently, the hydraulic impact on the central Nile Delta aquifer system is less severe than lining all canals throughout the study area.

#### Scenario 3: Zefta canals lining

For the Zefta canals lining scenario, groundwater recharge from canal seepage was reduced to 431,408 m^3^/day, corresponding to a decrease of approximately 28% from the base case scenario. Total inflow reached 2,892,935 m^3^/day, remaining relatively close to existing conditions. Discharge through the constant head boundaries declined slightly to 1,996,828 m^3^/day, while groundwater leakage back to canals was 6,617 m^3^/day. Compared with canal lining in Gharbia scenario, the influence on the regional groundwater zone budget was less pronounced, indicating that canals within the Zefta area contribute a smaller proportion of total seepage recharge. Therefore, lining only the Zefta canals preserves a greater share of aquifer recharge while still reducing conveyance losses.

#### Scenario 4: All canals covering (Gharbia and Zefta areas)

The all canals covering scenario completely eliminated seepage exchange between the canal network and the aquifer system. Stream leakage was reduced to zero, resulting in a total aquifer inflow of 2,501,188 m^3^/day, the lowest among all investigated scenarios. Correspondingly, groundwater discharge through the constant head boundaries reduced to 1,611,698 m^3^/day, while no groundwater outflow occurred through the stream boundaries. The complete removal of canal-aquifer interaction displays the maximum potential reduction in seepage losses; however, it also removes an important source of groundwater recharge to the aquifer system. This scenario is expected to produce the greatest reduction in groundwater levels and aquifer recharge within the study area.

#### Scenario 5: Gharbia canals covering

Covering canals within the Gharbia area only reduced groundwater recharge from canal seepage to 201,801 m^3^/day, which is nearly identical to the groundwater recharge reduction achieved by the Gharbia lining scenario. The total groundwater inflow declined to 2,647,617 m^3^/day, while discharge through the constant head boundaries reached 1,753,672 m^3^/day. Groundwater leakage toward canals reduced to 4,455 m^3^/day. These outcomes of this scenario indicate that canal covering in Gharbia is highly effective in minimizing seepage losses from canals and produces groundwater budget changes comparable to the canal lining. The similarity between the two scenarios indicates that both management measures achieve nearly equivalent hydraulic responses at the regional scale.

#### Scenario 6: Zefta canals covering

In the Zefta canals covering scenario, groundwater recharge from canals diminished to 423,501 m^3^/day, indicating a reduction of approximately 30% relative to the base case scenario. Total inflow was reduced to 2,886,993 m^3^/day, while discharge through the constant head boundaries decreased to 1,991,069 m^3^/day. Groundwater flow from the aquifer back to the canal network reached 6,433 m^3^/day. Similar to the Zefta lining scenario, the overall impact on the aquifer water budget was moderate, representing that the Zefta canal system contributes a smaller fraction of total recharge compared with the Gharbia canal network. Although canal covering reduced seepage slightly more than lining, the differences between the two management approaches remained relatively small.

#### Overall comparison of the six scenarios

The water budget analysis demonstrates that covering or lining all canals produced the largest reductions in groundwater recharge and total aquifer inflow, with all canals covering scenario generating the greatest impact by completely eliminating canal seepage. Management actions applied within the Gharbia area resulted in larger reductions in aquifer recharge than those implemented within the Zefta area, highlighting the greater hydraulic importance of the Gharbia canal network. In all scenarios, irrigation return recharge remained the dominant source of groundwater replenishment, while reductions in canal seepage were accompanied by corresponding decreases in groundwater discharge through the constant head boundaries. These findings indicate that canal rehabilitation measures can effectively reduce water losses from the conveyance system; however, they also decrease groundwater aquifer recharge.

#### Scenario No 1: canals lining (Gharbia and Zefta directorates canals lining)

**Fig. 17:**
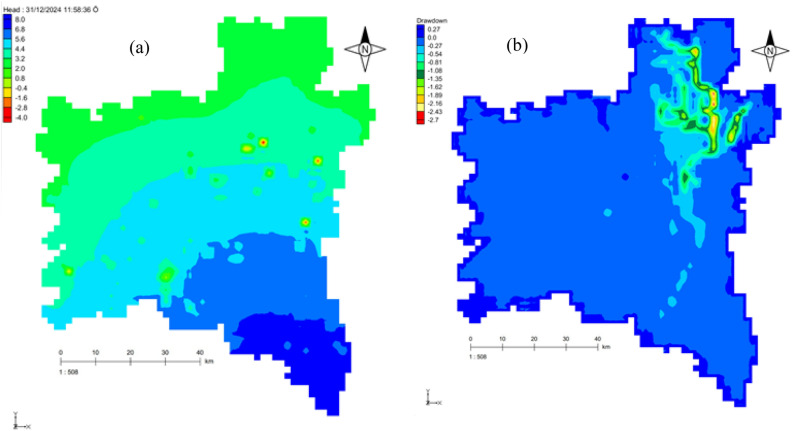
(**a**) Groundwater Head Distribution in the Central Part of the ND Region (Gharbia Governate) for (Gharbia and Zefta Directorates Canals Lining) for the year 2024 and (**b**) Drawdown for Scenario 1.

The research area’s groundwater levels were lowered because of canal lining, which decreased the aquifer net recharge from distribution canals. While taking into account that the lining’s execution will conclude in 2024. Overall, across the central part of the ND, the seepage from irrigation canals to the aquifer reached to 23920 m^3^/day, while groundwater flows to the canals from the aquifer at a rate of 368 m^3^/day. On the other hand, the groundwater aquifer discharge to the constant head borders declined to 1631585 m^3^/day, the net groundwater aquifer recharge from the constant head boundaries (the two Nile River beaches, Damietta and Rosetta) raised and accounted for 736149.9 m^3^/day. As a result of the simulated Damietta and Rosetta branches are not lined to compensate for the reduced channel infiltration into the aquifer. Likewise, the rise of groundwater to the south (upstream) is reduced because the canal lining indicates that the infiltration of the canal is reduced, as show in Fig. 17.

For scenario No. 1, the simulated groundwater level in the middle section of the ND (Gharbia governate) in 2024 ranged from 2 m in the north direction to 8 m in the south direction. The groundwater level drops to 3.2 meters in the west, 4.4 meters in the southwest, and 2 meters along the northern west boundary.

In a scenario analysis examining the effect of the canal lining on the variations of the groundwater levels for the Walla Walla Basin, USA, Scherberg et al.^62^ found comparable outcomes. Similarly, Meijer et al.^63^ demonstrated that the concrete lining the irrigation canals considerably declined the elevation of the groundwater table, which in turn impacted the abstraction of water from shallow wells for residential use. Only 0.9% of the total inflow comes from aquifer seepage through canals, and canal outflow from the aquifer is minimal, which is acceptable^55^.

The drawdown in the central part of the ND region (Gharbia governate) ranged between (0.0) to (−2.43) m. Most of the governate, drawdown ranged between (0.0) to (−0.27) m. The minimum drawdown observed in the southern, western and central region of the study area and ranged from (0.0) to (−0.27). The maximum drawdown observed in the northern and northeast region of the study area and varied from (−0.54) to (−2.43) m, as shown in Fig. 17.

Lining all irrigation canals significantly changed the groundwater budget by reducing seepage from 602,081 m^3^/day in the base case to only 23,921 m^3^/day, demonstrating a reduction of approximately 96%. Because canal seepage establishes an important source of aquifer replenishment, this reduction resulted in a noticeable decline in groundwater levels through the study area. The reduction in groundwater recharge was partially compensated by increased inflow from the Rosetta and Damietta branches, demonstrating a stronger hydraulic connection between the aquifer system and the river boundaries.

The groundwater head distribution continued generally consistent with the regional groundwater flow system; however, lower hydraulic heads were observed compared with the base case. The resulting drawdown changed from negligible values in the southern sector to a maximum of 2.43 m in the northern and northeastern parts of the study area. These areas experienced the largest impact because they are located farther from major recharge sources and are more dependent on canal seepage. The outcomes displayed that large-scale canal lining can substantially improve water conveyance efficiency but may adversely affect the groundwater storage and the central Nile Delta aquifer sustainability.

#### Scenario No 2: lining of Gharbia directorate canals only

**Fig. 18:**
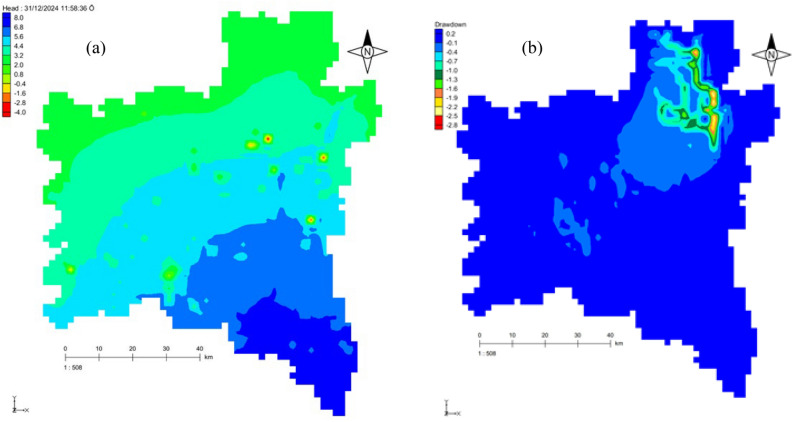
(**a**) Groundwater Heads Distribution in the Central Part of the ND Region (Gharbia Governate) for Lining of Gharbia directorate Canals Only for the year 2024 and (**b**) Drawdown for Scenario 2.

Nearly 127 canals need to be lined by Gharbia’s General Directorate of Irrigation. Overall, the groundwater flows from the aquifer system to the irrigation canals reached 4633 m^3^/day, while canal seepage to the aquifer reached 203881 m^3^/day. On the other hand, the groundwater aquifer discharge to the constant head borders declined to 1761341 m^3^/day, the net groundwater aquifer recharge from the constant head boundaries (the two branches of the Nile, Rosetta and Damietta) increased and accounted for 684804 m^3^/day. The simulated groundwater potentiometric head distributions for Gharbia canal lining are shown in Fig. 18. For scenario No. 2, the simulated groundwater level in the middle section of the ND (Gharbia governate) in 2024 was 2 m in the north direction and rose to 8 m in the south direction. While the groundwater level is 4.4 meters in the southwest and drops to 2 meters in the northwest, it drops to 3.2 meters in the west. The drawdown in the central part of the ND region (Gharbia governate) ranged between (−0.1) to (−2.5) m. Most of the governate, drawdown ranged between (−0.1) to (−0.4) m. The maximum drawdown observed in the northern and the northeast region and varied from (−0.7) to (−2.5 ) m. The minimum drawdown observed in the southern and the southwest region and equaled (-0.1) m, as presented in Fig. 18.

The lining of canals within the Gharbia irrigation district declined seepage recharge to 203,881 m^3^/day, corresponding to a reduction of approximately 66% relative to the base case. While the reduction was less severe than in Scenario 1, groundwater levels still declined across a considerable portion of the study area. The reduced seepage recharge decreased groundwater discharge toward the constant-head boundaries and altered the regional groundwater balance. The drawdown pattern showed a maximum decline of about 2.5 m, which was slightly greater than that observed under Scenario 1. The largest groundwater-level declines occurred in the northern and northeastern sectors, whereas southern areas displayed only minor changes. This response reveals that the Gharbia canal network contributes significantly to groundwater recharge in the central Nile Delta aquifer system. Accordingly, rehabilitation measures employed within Gharbia have a greater impact on aquifer conditions than similar interventions in other parts of the study area.

#### Scenario No 3: lining of Zefta directorate canals only

**Fig. 19:**
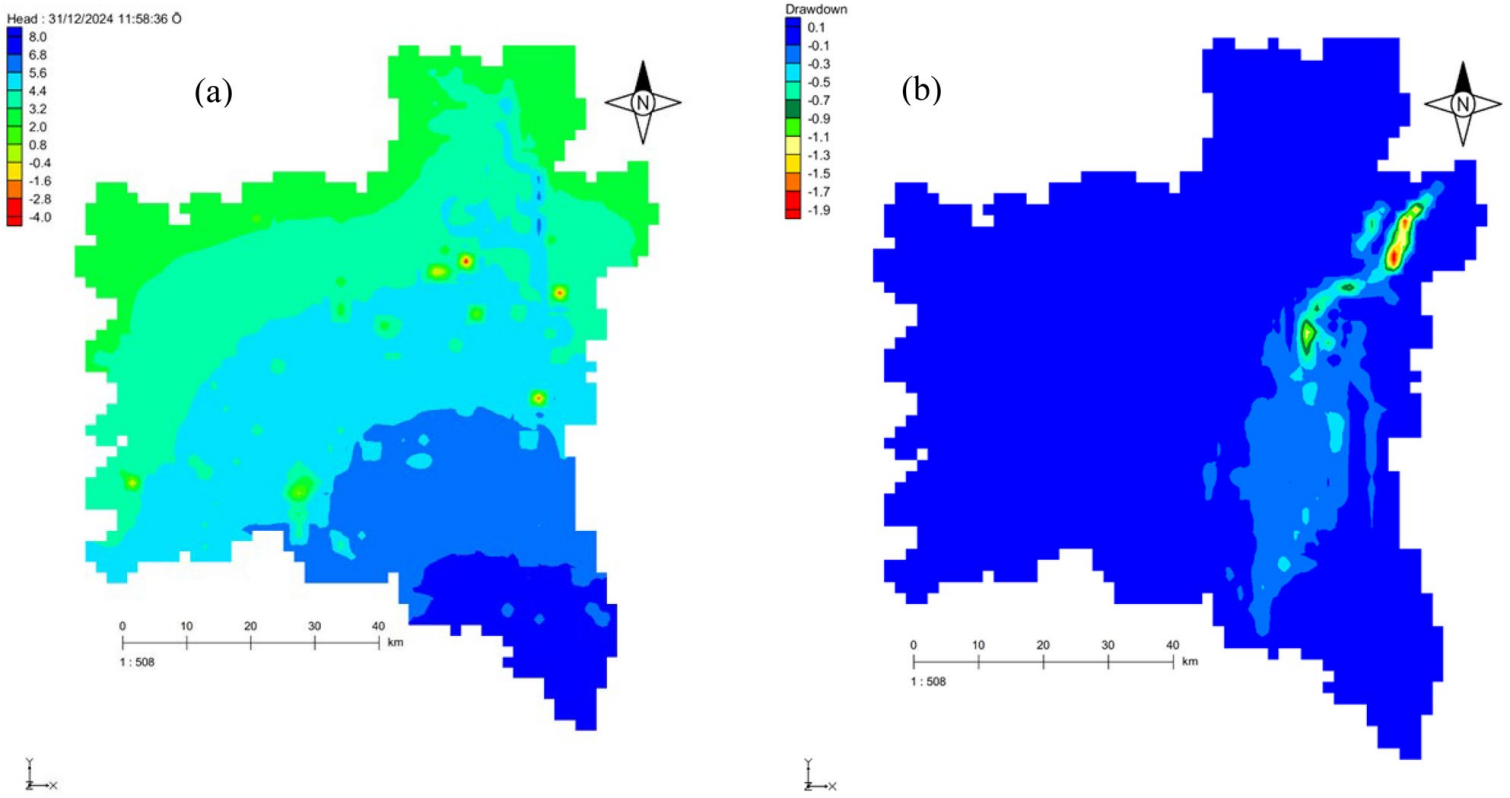
(**a**) Groundwater Heads Distribution in the Central Part of the ND Region (Gharbia Governate) for Lining of Zefta Directorate Canals Only for the year 2024, and (**b**) Drawdown for Scenario 3.

Nearly 97 canals need to be lined by Zefta’s General Directorate of Irrigation. Overall, the groundwater flows from the groundwater aquifer system to the canals totaled 6617 m^3^/day, while canal seepage to the aquifer reached 431408 m^3^/day. On the other hand, the groundwater aquifer discharge to the constant head borders declined to 1996828 m3/day, the net groundwater aquifer recharge from the constant head boundaries (the two branches of the Nile, Rosetta and Damietta) increased and accounted for 700169 m^3^/day. The simulated groundwater potentiometric head distributions for Zefta canal lining are shown in Fig. 19. For scenario No. 3, the simulated groundwater level in the middle portion of the ND (Gharbia governate) in 2024 ranged from 2 m in the northern direction to 8 m in the southern direction. The groundwater level dropped to 3.2 meters in the west, 4.4 meters in the southwest, and 2 meters in the northwest. The drawdown in the central part of the ND region (Gharbia governate) varied between (−0.1) to (−1.7) m. Most of the governate, drawdown ranged between (0.0) to (−0.1) m. The maximum drawdown observed in the northeast, eastern and southern region and ranged from (−0.3) to (−1.7) m. The minimum drawdown observed in the northern, western and central region and equaled from (-0.1) m, as presented in Fig. 19.

Restricting canal lining to the Zefta irrigation district produced a more moderate influence on the groundwater system. Canal seepage recharge reduced to 431,408 m^3^/day, indicating a reduction of about 28% compared with the base case. As a result, groundwater heads experienced relatively limited declines, and the overall groundwater flow regime remained largely unchanged. The maximum observed drawdown reached approximately 1.7 m, which was significantly smaller than the values obtained in Scenarios 1 and 2. Most of the study area, in the central of the Nile Delta, experienced only minor groundwater-level reductions, generally less than 0.1 m. The greatest impacts were concentrated on the eastern and northeastern portions of the study area. These outcomes suggested that canals within the Zefta district contribute less groundwater recharge to the regional aquifer system than those within Gharbia, making this scenario less disruptive to groundwater resources.

#### Scenario No 4: covering canals (Gharbia and Zefta directorates canals covering)

**Fig. 20:**
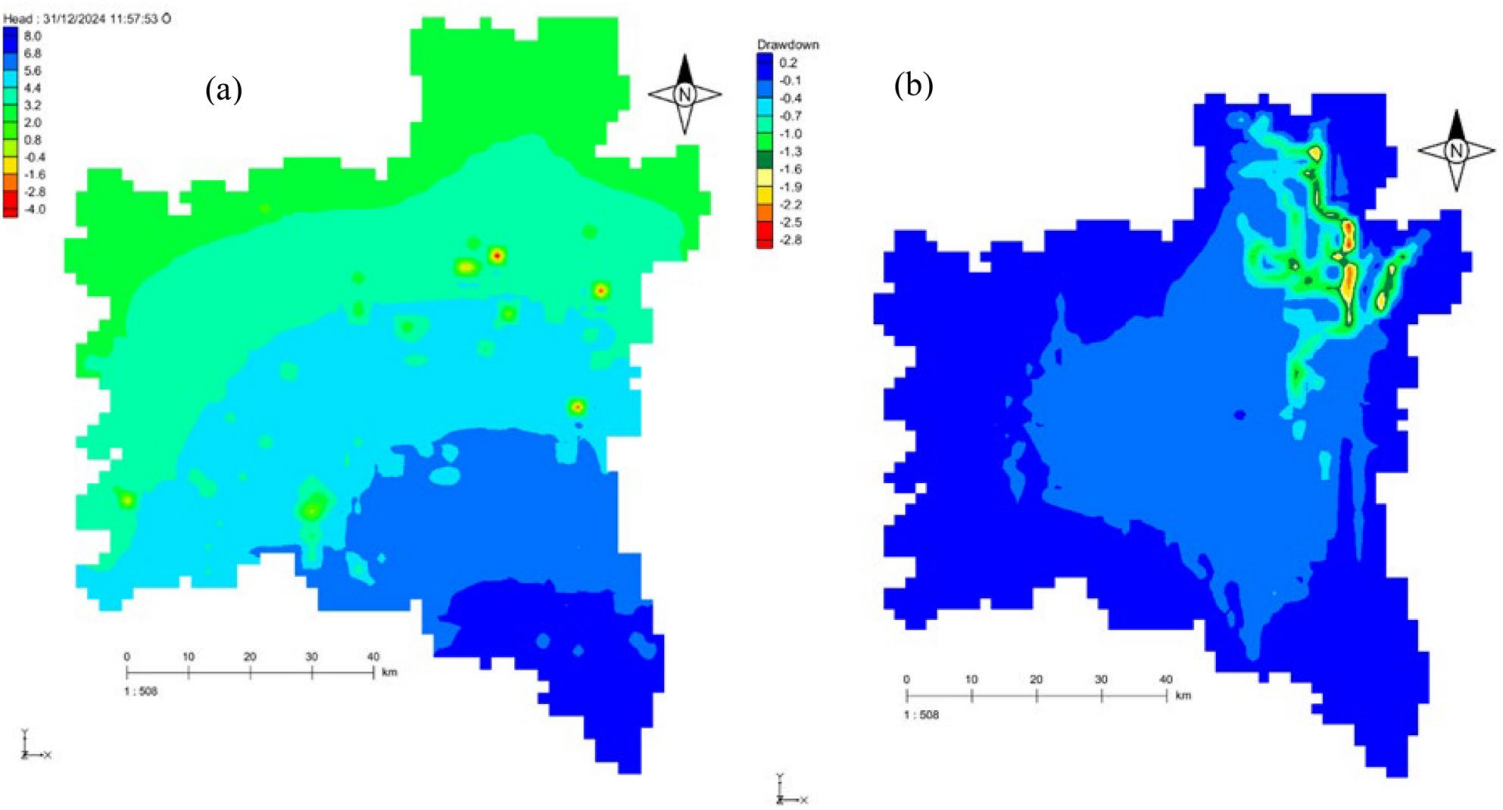
(**a**) Groundwater Head Distribution in the Central Part of the ND Region (Gharbia Governate) for (Gharbia and Zefta Directorates Canals Covering) for the year 2024, and (**b**) Drawdown for Scenario 4.

In contrast to the prior scenario, groundwater was reduced when the canals were covered; only approximately 0.0603 m^3^/day leaked out of the canals to the groundwater aquifer system, and the canals did not receive any groundwater recharge from the aquifer. It is evident from the inflow parameters table 5-1 that the main groundwater recharge to the aquifer in these circumstances is the return flow from irrigation water. In contrast to irrigation water recharge, canal seepage makes a negligible contribution to the aquifer. The contribution of the seepage of irrigation canals to the aquifer in the current scenario (basic case without lining or covering) was 19.96%. The main outflow parameter is groundwater extractions, which accounts for 29.5% of the aquifer discharge. The simulated groundwater potentiometric head distributions for the scenario of (Gharbia and Zefta Directorates Canals Covering) are shown in Fig. 20. For scenario No. 4, the simulated groundwater level in the middle section of the ND (Gharbia governate) in 2024 was 2 m in the northern direction and rose to 8 m in the southern direction. The groundwater level dropped to 3.2 meters in the west, 4.4 meters in the southwest, and 2 meters in the northwest. The drawdown in the central part of the ND region (Gharbia governate) ranged between (−0.1) to (−2.5) m. Most of the governate, drawdown varied between (−0.1) to (−0.4) m. The maximum drawdown observed in the northern and northeast region and ranged from (−0.7) to (−2.5) m. The minimum drawdown observed in the western region and equaled (−0.1), as shown in Fig. 20.

Covering all canals produced the most substantial modification of the groundwater budget among all investigated scenarios. Canal seepage was effectively eliminated, removing a major source of groundwater aquifer recharge. Accordingly, groundwater replenishment became increasingly dependent on irrigation return flow and river-boundary inflows. The reduction in groundwater recharge resulted in widespread groundwater-level declines throughout the study area. The drawdown pattern closely resembled that of Scenario 2 but displayed a slightly greater spatial extent. Maximum drawdown values approached 2.5 m in the northern and northeastern sectors, while southern areas experienced comparatively small groundwater declines. The outcomes indicated that although canal covering maximizes water-saving benefits by minimizing canal seepage and evaporation losses, it also produces the greatest adverse impact on the groundwater resources in the study area. Consequently, widespread canal covering may not be appropriate in areas where groundwater recharge is required to maintain aquifer sustainability.

#### Scenario No 5: covering canals (Gharbia Directorate canals covering only)

**Fig. 21:**
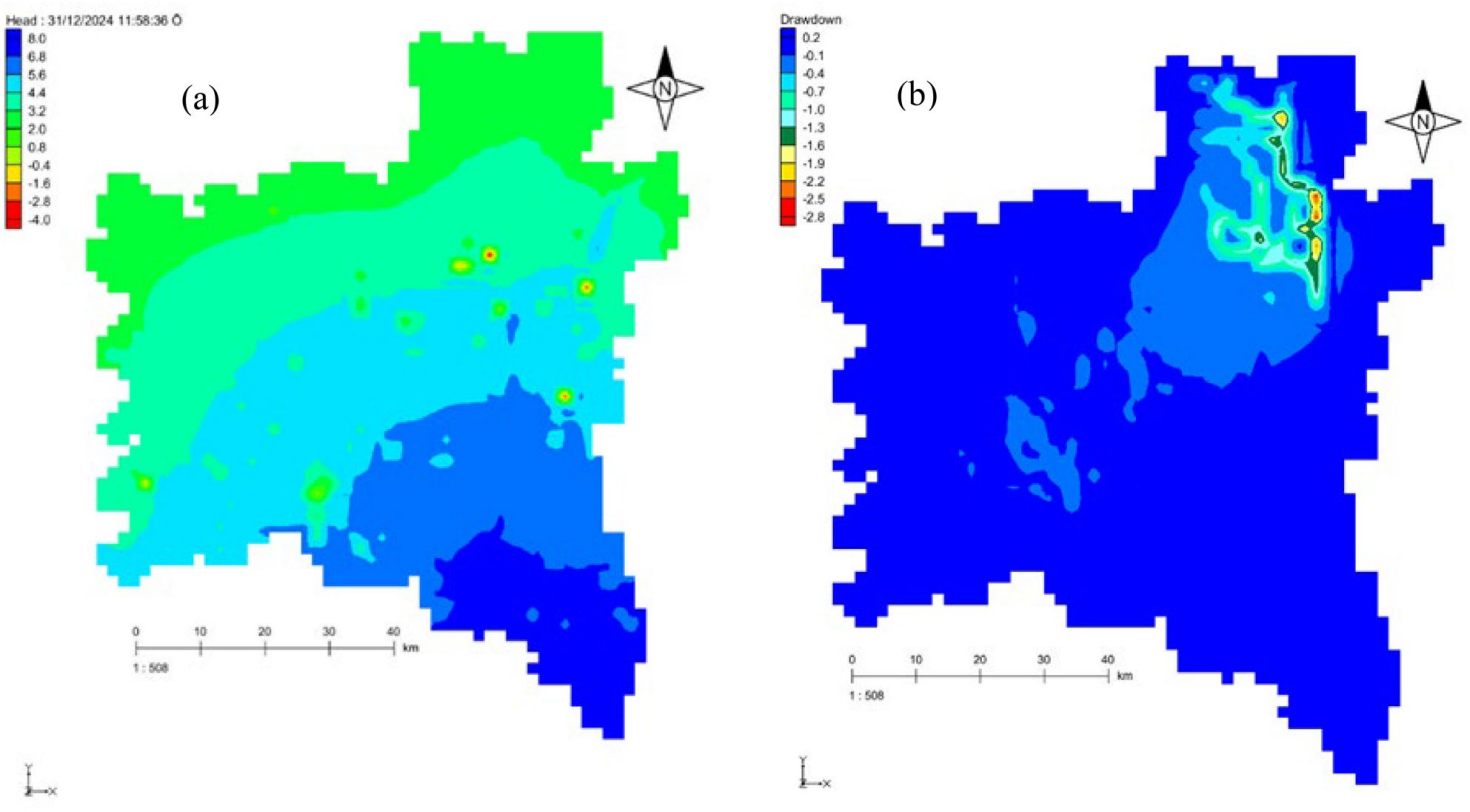
(**a**) Groundwater head distribution in the central part of the ND region (Gharbia governate) for (Gharbia directorate canals covering only for the year 2024, and (**b**) Drawdown for Scenario 5.

We assumed enclosing the irrigation canals in conduits in General Directorate of Irrigation in Gharbia and was simulated in a model. While canals get recharge from the aquifer at a rate of 4455 m^3^/day, only around 201801 m3/day seeped out of the canals to the aquifer. On the other hand the groundwater aquifer discharge to the constant head boundaries was reduced to 1753672 m^3^/day, the net groundwater aquifer recharge from the two Nile River beaches, Damietta and Rosetta, raised to 684451 m^3^/day. The simulated groundwater potentiometric head distributions for Gharbia directorate canals covering only scenario are shown in Fig. 21. For scenario No. 5, the simulated groundwater level in the center portion of the ND (Gharbia governate) in 2024 varied from 2 m in the northern direction to 8 m in the southern direction. While the groundwater level is 4.4 meters in the southwest and drops to 2 meters in the northwest, it drops to 3.2 meters in the west. The drawdown in the central part of the ND region (Gharbia governate) ranged between (−0.1) to (−2.5) m. Most of the governate, drawdown ranged between (0.0) to (−0.1) m. The maximum drawdown observed in the northern and northeast region and varied from (−0.4) to (−2.5) m. The minimum drawdown observed in the northern and western region and equaled (−0.1) m, is presented in Fig. 21.

Covering canals within the Gharbia irrigation district reduced canal recharge to approximately 201,801 m^3^/day, which is nearly identical to the reduction achieved through canal lining in the same area. Consequently, the groundwater response was also similar. Lower groundwater recharge rates reduced the groundwater storage and produced drawdown over much of the study area. The maximum simulated drawdown reached approximately 2.5 m, primarily within the northern and northeastern regions. On the other hand, groundwater levels in the southern and western sectors remained relatively stable. The similarity between Scenarios 2 and 5 indicated that both lining and covering of Gharbia canals produce comparable impacts on the groundwater system. This finding suggests that the reduction of canal seepage itself, rather than the specific rehabilitation method, is the primary factor controlling groundwater response.

#### Scenario No 6: covering canals (Zefta directorate canals covering) only

**Fig. 22:**
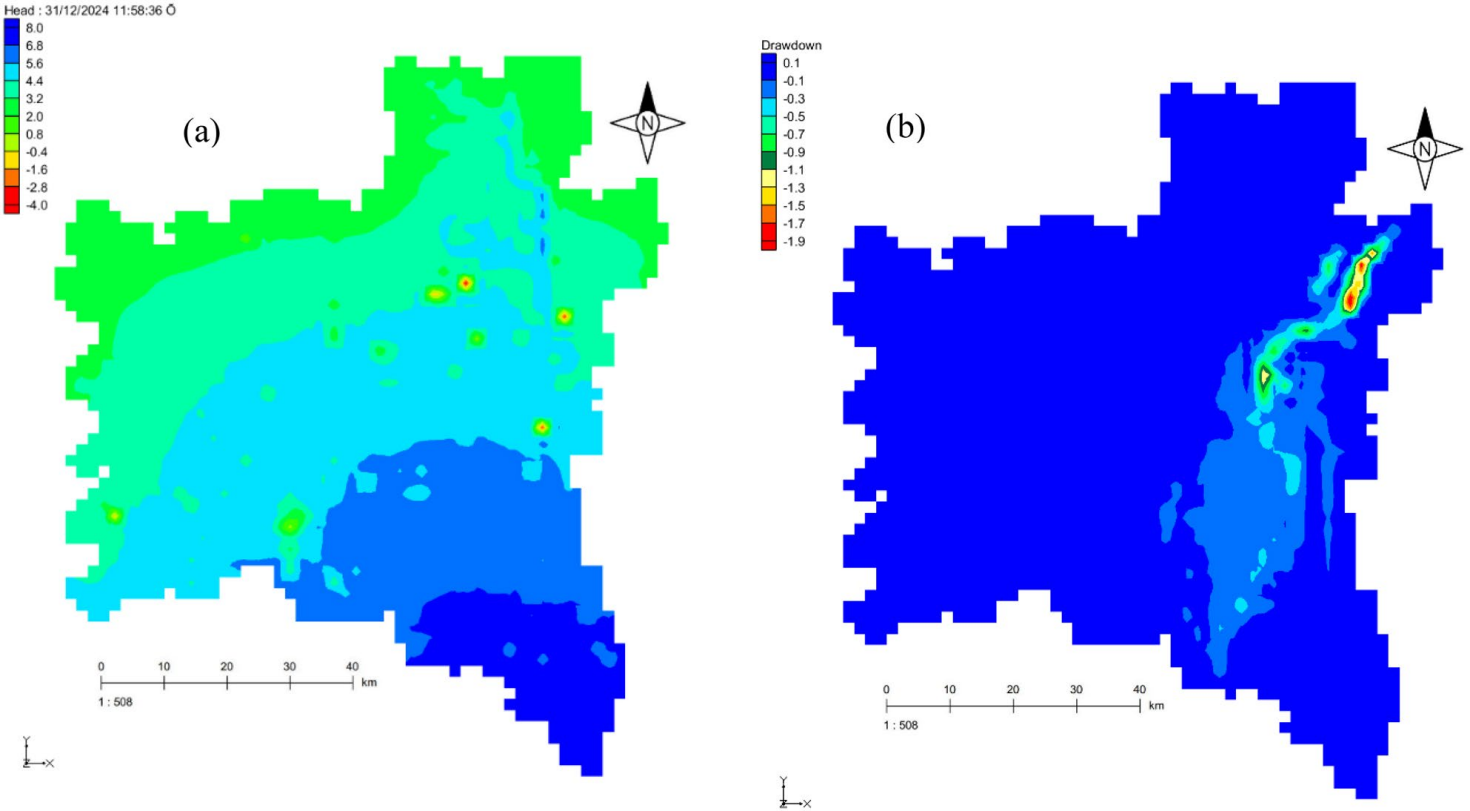
(**a**) Groundwater head distribution in the central part of the ND region for Zefta Directorate canals covering only for the year 2024, and (**b**) Drawdown for Scenario 6.

We assumed enclosing the distribution canals in conduits in General directorate of irrigation in Zefta and was simulated in a model. Canals get roughly 6433 m^3^/day of recharge from the aquifer, but only about 423501 m^3^/day were seeped out to the aquifer. The groundwater aquifer discharge to the constant head boundaries was raised to 1991069 m^3^/day, on the other hand the net groundwater aquifer recharge from the constant head boundaries (the two Nile River beaches, Damietta and Rosetta) was increased and accounted for 702133 m^3^/day. The simulated groundwater potentiometric head distributions for Zefta directorate canals covering only scenario are shown in Fig. 22. For scenario No. 6, the simulated groundwater level in the middle section of the ND (Gharbia governate) in 2024 was 2 m in the northern direction and rose to 8 m in the southern direction. While the groundwater level is 4.4 meters in the southwest and drops to 2 meters in the northwest, it drops to 3.2 meters in the west. The drawdown in the central part of the ND region (Gharbia governate) varied between (−0.1) to (−1.9) m. Most of the governate, drawdown ranged between (0.0) to (−0.1) m. The maximum drawdown observed in northeast and eastern region and ranged from (-0.3) to (−1.9) m. The minimum drawdown observed in northern, western and central region and equaled (−0.1) m, is shown in Fig. 22. The covering of canals within the Zefta irrigation district produced a moderate reduction in groundwater recharge, decreasing canal seepage to 423,501 m^3^/day. However recharge losses were evident, the groundwater system preserved a substantial portion of its original replenishment capacity because much of the canal network remained unchanged. Groundwater-level declines were less pronounced than those detected in the Gharbia-focused scenarios. Maximum drawdown reached approximately 1.9 m and was concentrated mainly in the eastern and northeastern sectors of the study area. Most locations experienced only minor groundwater-level changes. Compared with Scenarios 1, 2, 4, and 5, this scenario produced a considerably smaller impact on groundwater storage, representing that the Zefta canal network contributes a smaller proportion of total seepage recharge to the central Nile Delta aquifer.

The six management scenarios confirmed a clear relationship between canal seepage reduction and groundwater-level decline. The most severe impact occurred when all canals were covered (Scenario 4) or lined (Scenario 1), as these measures removed nearly all seepage-derived recharge. Intermediate influences were observed when rehabilitation was limited to the Gharbia irrigation district (Scenarios 2 and 5), while the smallest impacts resulted from interventions restricted to the Zefta district (Scenarios 3 and 6). Comparison of the drawdown maps showed that the northern and northeastern portions of the central Nile Delta are the most sensitive areas to reductions in canal seepage. Maximum groundwater-level declines ranged from approximately 1.7 m under Scenario 3 to about 2.5 m under Scenarios 2, 4, and 5. The outcomes highlight the dual role of canal seepage as both a water loss mechanism and an important source of aquifer recharge. Therefore, although canal lining and covering improves irrigation conveyance efficiency, they also reduce groundwater replenishment and may negatively influence long-term aquifer sustainability. Effective water-management strategies should balance the benefits of water conservation against the need to maintain adequate groundwater recharge.

#### Estimation of seepage losses from the canals using analytical equations and empirical formulas

A comparison between the seepage rates from the canals in Gharbia irrigation directorate and Zefta irrigation directorate estimated using empirical formulas, analytical equations, with data and information gave by the Gharbia & Zefta irrigation directorates.

The total seepage loss figures in the canals are displayed in Table 5. This makes it evident that: The seepage loss values determined using the most often used equations above differ reasonably. For the empirical formulas, David&Wilson formula gives the biggest value, while for analytical equations, Vedernikov gives the biggest values, for canals network.

**Table 5:** The Values of Seepage Losses in the Canals by Analytical, Empirical Methods and GMS (values in m^3^/year).

Solution	Equations	Seepage losses x 10⁹ (m^3^/year)
Empirical formulas	David&Wilson	1.08
	The Egyptian formula	0.97
	Pakistanian formula	0.93
	Hungarian formula	0.03
	Mowafy	0.33
	Moritz	0.56
	Ingham	0.62
	Nazir Ahmed	0.03
Analytical equations	Molesworth& Yennidunia equation	0.24
Numerical model	GMS	0.22

For The empirical formulas, Nazir Ahmed formula gives the lowest value, while for analytical equations, Molesworth and Yennidunia gives the lowest values, for the canals network.

The total amount of seepage losses from the sections of canals estimated by using Davis-Wilson, the Egyptian formula, Pakistanian, Hungarian, Mowfey, Moritz, Ingam, Nazir

Ahmed, Molesworth and Yennidunia, and GMS represents about (1.08 x 10^9^), (0.97x10^9^), (0.93 x 10^9^),(0.03 x 10^9^), (0.33 x 10^9^), (0.56 x 10^9^), (0.62 x 10^9^), (0.03 x 10^9^), (0.24 x 10^9^), and (0.22 x 10^9^) m^3^/year, respectively Fig. 19.

**Fig. 23:**
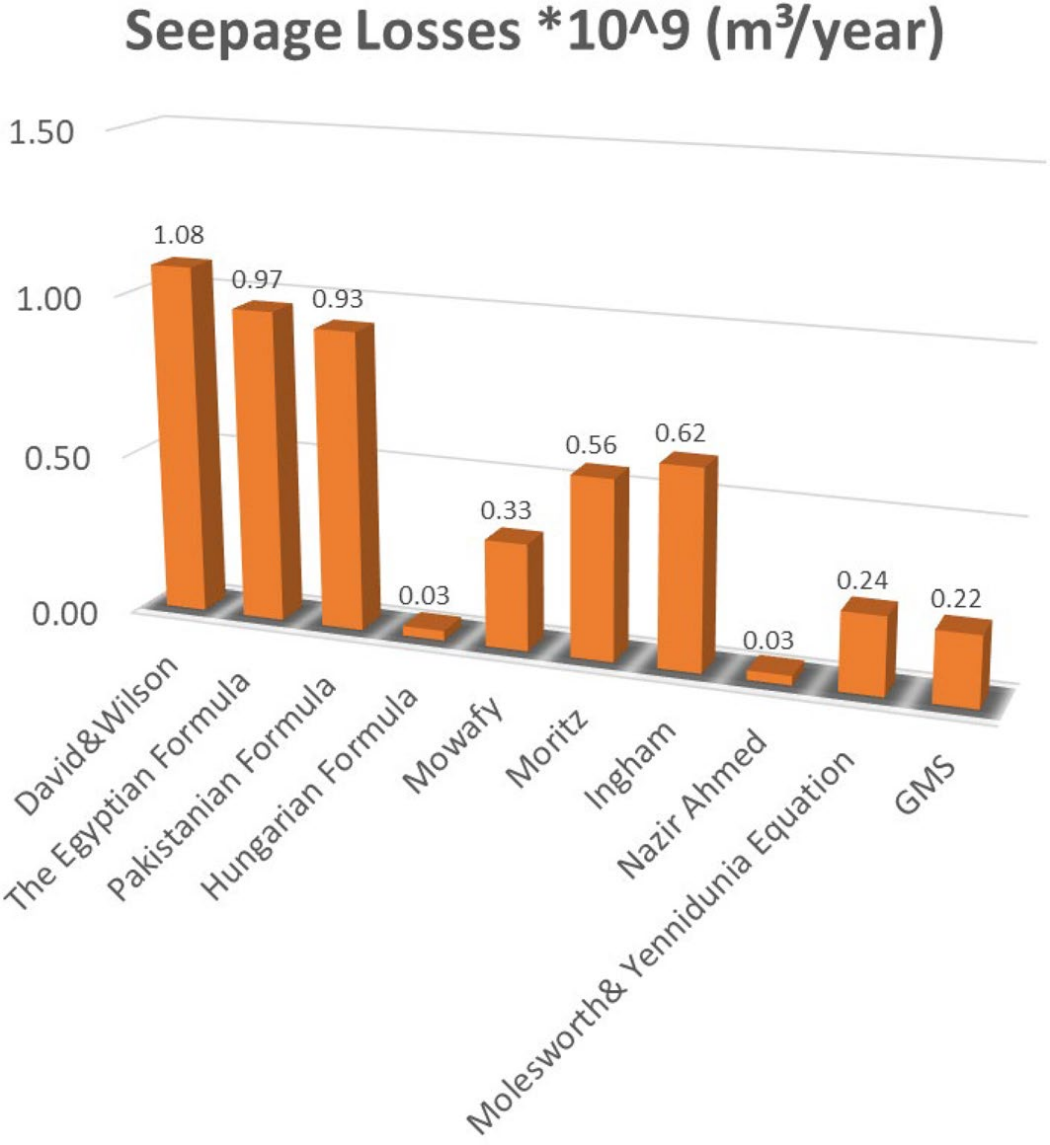
Canal seepage losses from the earthern canals using empirical formulas, analytical formulas, and GMS.

As illustrated in Fig. 23, seepage losses were calculated and compared using a variety of analytical and empirical techniques to validate the numerical model.

**Fig. 24:**
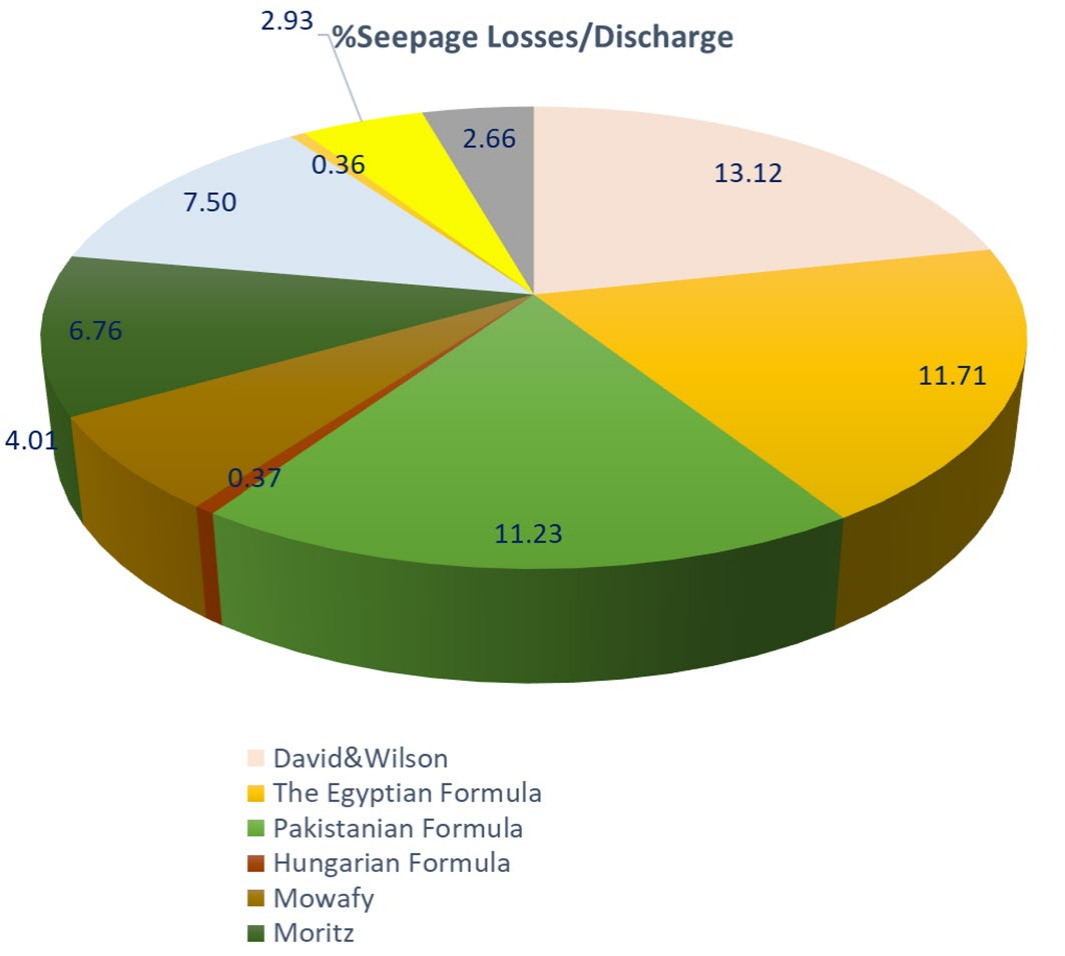
The Percentage of seepage losses to the canals discharge.

Figure 24 shows the percentage of seepage to the canals discharge in Davis-Wilson, the Egyptian formula, Pakistanian, Hungarian, Mowfey, Moritz, Ingam, Nazir Ahmed, Molesworth and Yennidunia, and GMS which is estimated about (13.12), (11.71), (11.23), (0.37), (4.01), (6.76), (7.50), (0.36), (2.93), (2.66) %, respectively.It is evident that the quantitatively determined amount of water seepage (0.22 x 10^9^) m^3^/year in this study agrees very well with the analytical solutions of Molesworth and Yennidunia (0.24 x 10^9^) m^3^/year. This observation is in agreement with^64^ recommendations for using this method to determine seepage losses in Egyptian earthen canals.

#### Comparative evaluation of canal rehabilitation scenarios

Table 6 shows the canal seepage values and percentage of reduction for the studied scenarios compared with the base case. The six management scenarios produced significantly various influences on groundwater recharge and groundwater levels in the study area. The greatest reduction in groundwater aquifer recharge occurred when all canals were covered (Scenario 4), followed by lining all canals (Scenario 1). Both measures substantially decreased seepage from the irrigation network, producing widespread groundwater-level declines throughout the study area. Partial implementation within the Gharbia irrigation district (Scenarios 2 and 5) produced intermediate impacts, reducing canal seepage recharge by approximately two-thirds relative to the base case. On the other hand, the Zefta-only interventions (Scenarios 3 and 6) resulted in smaller changes because the canals within this district contribute a smaller fraction of total seepage recharge. The drawdown maps show that the northern and northeastern portions of the study area are the most sensitive to reductions in canal seepage. These areas consistently exhibited the largest groundwater-level declines under all scenarios. The maximum simulated drawdown reached approximately 2.5 m under Scenarios 2, 4, and 5, whereas the smallest impacts were observed under Scenario 3, where maximum drawdown remained below 1.7 m. This spatial pattern reflects the dependence of northern areas on recharge supplied by the canal network. Overall, the outcomes demonstrate a clear trade-off between irrigation conveyance effectiveness and groundwater sustainability. Measures that minimize seepage losses improve water delivery efficiency but simultaneously reduce aquifer replenishment and groundwater storage.

**Table 6:** Canal seepage values and percentage of reduction for the studied scenarios compared with the base case.

Scenario	Canal seepage (m^3^/day)	Reduction from base case (%)
Base case	602,081	-
Scenario 1	23,921	96.0%
Scenario 2	203,881	66.1%
Scenario 3	431,408	26.4%
Scenario 4	0	−100%
Scenario 5	201,801	66.5%
Scenario 6	423,501	29.7%

The outcomes reveal an important management trade-off in the central Nile Delta. Canal lining and canal covering improve irrigation efficiency by reducing conveyance losses; however, these losses currently function as a major source of groundwater recharge. Consequently, interventions designed to conserve surface water also decrease the volume of water entering the groundwater aquifer system. Under the base-case conditions, canal seepage contributes approximately 20% of total groundwater recharge. Eliminating or reducing this recharge source causes groundwater levels to decline and increases the dependence of the aquifer on irrigation return flow and river-boundary inflows. Though groundwater abstraction remained constant in all simulations, reduced recharge altered the groundwater balance and generated drawdown through the study area. From a water-management perspective, complete canal rehabilitation may maximize water-delivery efficiency but could negatively affect shallow groundwater availability, particularly in areas that rely on groundwater for supplemental irrigation. Therefore, future rehabilitation programs should consider both surface-water savings and groundwater sustainability. A balanced strategy combining selective canal rehabilitation, groundwater monitoring, and managed aquifer recharge may provide greater long-term benefits than widespread implementation of impermeable canal systems.

The simulation results show that canal seepage represents an important component of the groundwater budget in the central Nile Delta aquifer system. Rehabilitation measures that reduce seepage substantially decrease groundwater recharge and generate measurable groundwater-level declines. Among the investigated alternatives, complete canal covering produced the greatest reduction in groundwater recharge, while interventions limited to the Zefta irrigation district had the smallest influence on groundwater conditions.

The outcomes demonstrate that improvements in irrigation conveyance efficiency are accompanied by reductions in groundwater aquifer replenishment. Therefore, canal rehabilitation strategies should be evaluated not only in terms of surface-water conservation but also with respect to their impact on groundwater resources. Integrated management approaches that account for both components of the hydrologic system are essential for achieving sustainable water-resource management in the central Nile Delta.

## Conclusion

The groundwater system of the central Nile Delta is strongly influenced by agricultural activities, with irrigation return flow acting as the primary source of groundwater aquifer replenishment. This dominance reflects the intensive irrigation practices in the region, where a substantial portion of applied irrigation water percolates through the soil profile and eventually recharges the shallow aquifer. In contrast, canal seepage represents a secondary but still important recharge mechanism that helps sustain groundwater levels, particularly in areas located away from the Nile branches. The simulation results demonstrate that reducing canal seepage through lining or covering measures improves irrigation water-conveyance efficiency but simultaneously decreases groundwater recharge. This finding highlights a critical trade-off within the water resources system of the central Nile Delta. While canal rehabilitation reduces water losses from the irrigation network, it also diminishes a source of aquifer replenishment that contributes to maintaining groundwater storage and hydraulic stability.

Comparison of the six management scenarios revealed that interventions applied across the entire canal network produced the greatest groundwater-level declines, whereas measures limited to the Zefta irrigation district generated the smallest impacts. The largest drawdowns consistently occurred in the northern and northeastern parts of the study area, indicating that these locations are particularly sensitive to reductions in canal-derived recharge. The results further showed that rehabilitation measures implemented within the Gharbia irrigation district had a greater influence on groundwater conditions than similar interventions in the Zefta district, reflecting the larger contribution of the Gharbia canal network to regional recharge processes. From a management perspective, the findings suggest that maximizing irrigation efficiency should not be considered independently from groundwater sustainability. Complete elimination of seepage losses may improve surface-water delivery performance, but it can also reduce aquifer replenishment and increase the vulnerability of groundwater resources in areas that depend on shallow groundwater. Therefore, future canal rehabilitation programs should adopt an integrated approach that evaluates both surface-water savings and groundwater impacts. Selective rehabilitation of high-loss canal reaches, combined with continuous groundwater monitoring and conjunctive management of surface water and groundwater resources, may provide a more balanced and sustainable solution. Overall, the study demonstrates that canal seepage functions not only as a conveyance loss but also as a hydrologically significant component of the groundwater recharge system in the central Nile Delta. Sustainable water-management strategies should therefore seek to balance improvements in irrigation efficiency with the long-term maintenance of aquifer recharge and groundwater availability, supporting regional water security and the objectives of Egypt Vision 2030.

## Data Availability

The data is available on request to the corresponding author.
